# Supplementary material for: Spatially resolved analyses link genomic and immune diversity and reveal unfavorable neutrophil activation in melanoma
Source: Nat Commun. 2020 Apr 15;11:1839. doi: 10.1038/s41467-020-15538-9 (PMC7160105; doi:10.1038/s41467-020-15538-9)
Supplement: Supplementary file 1 — Supplementary Information [file 41467_2020_15538_MOESM1_ESM.docx]

# Spatially resolved analyses link genomic and immune diversity and reveal unfavorable neutrophil activation in melanoma.

## A. Mitra, M.C. Andrews, *et al*.

**SUPPLEMENTARY INFORMATION**

# Supplementary Figure 1.

**Section 1B**


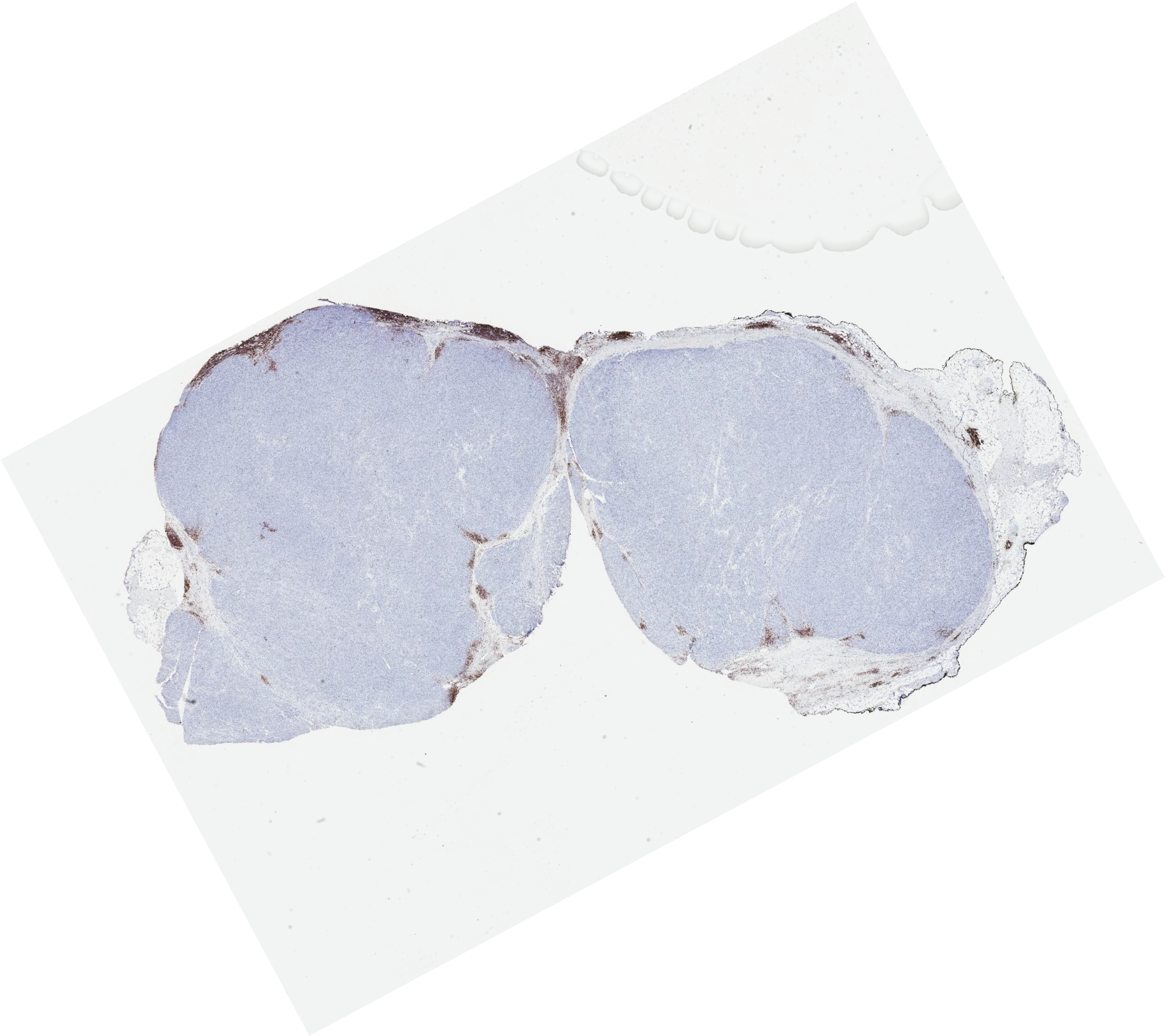

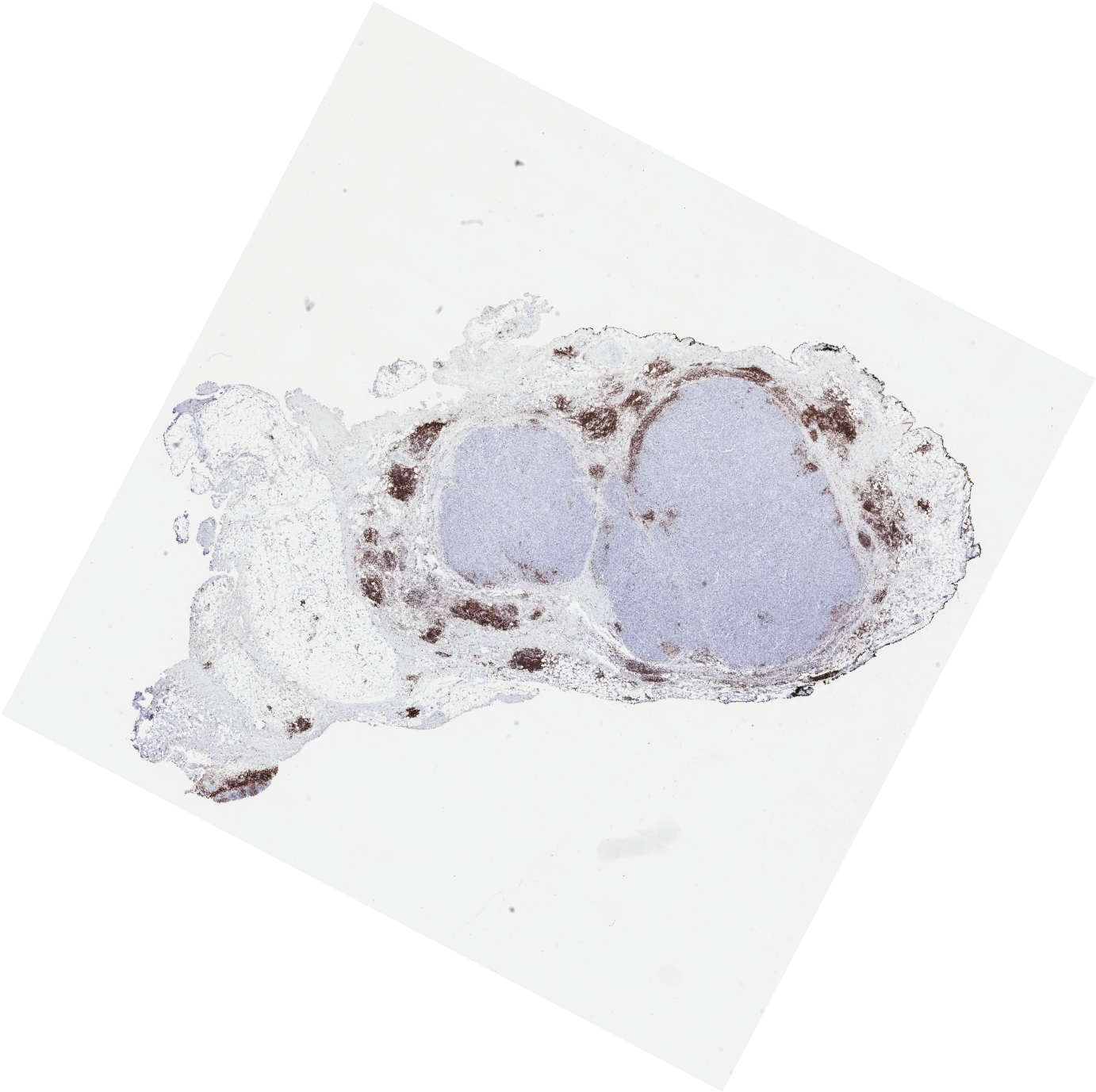

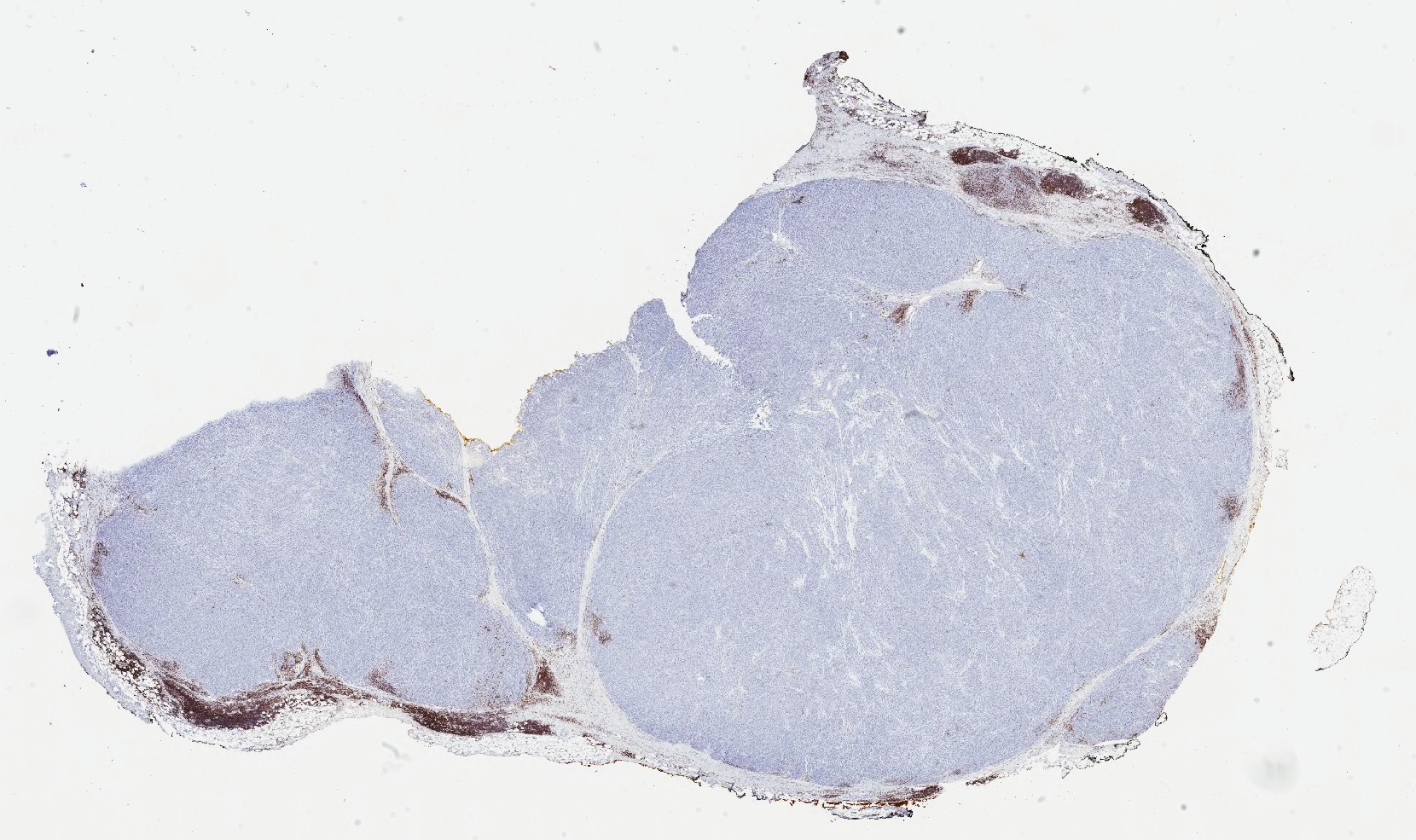

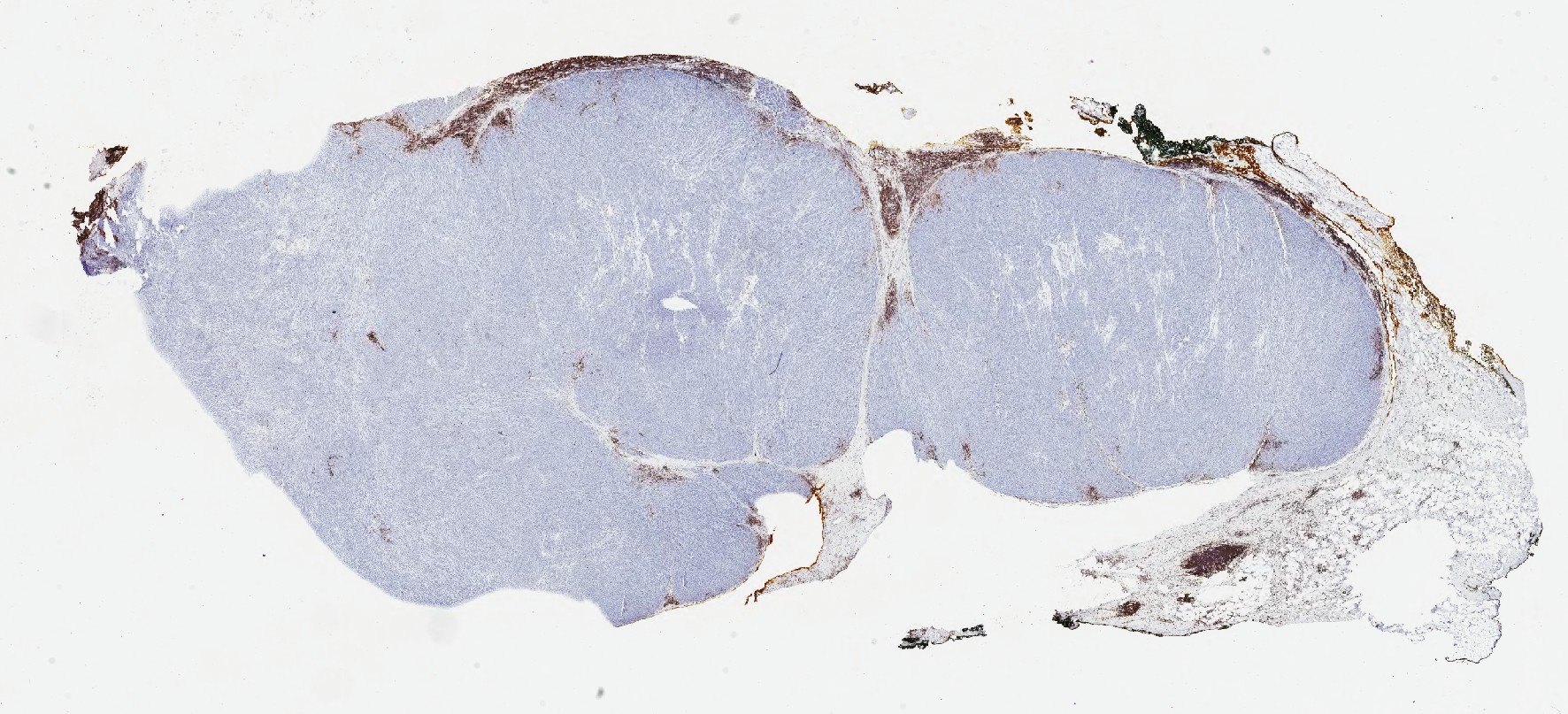

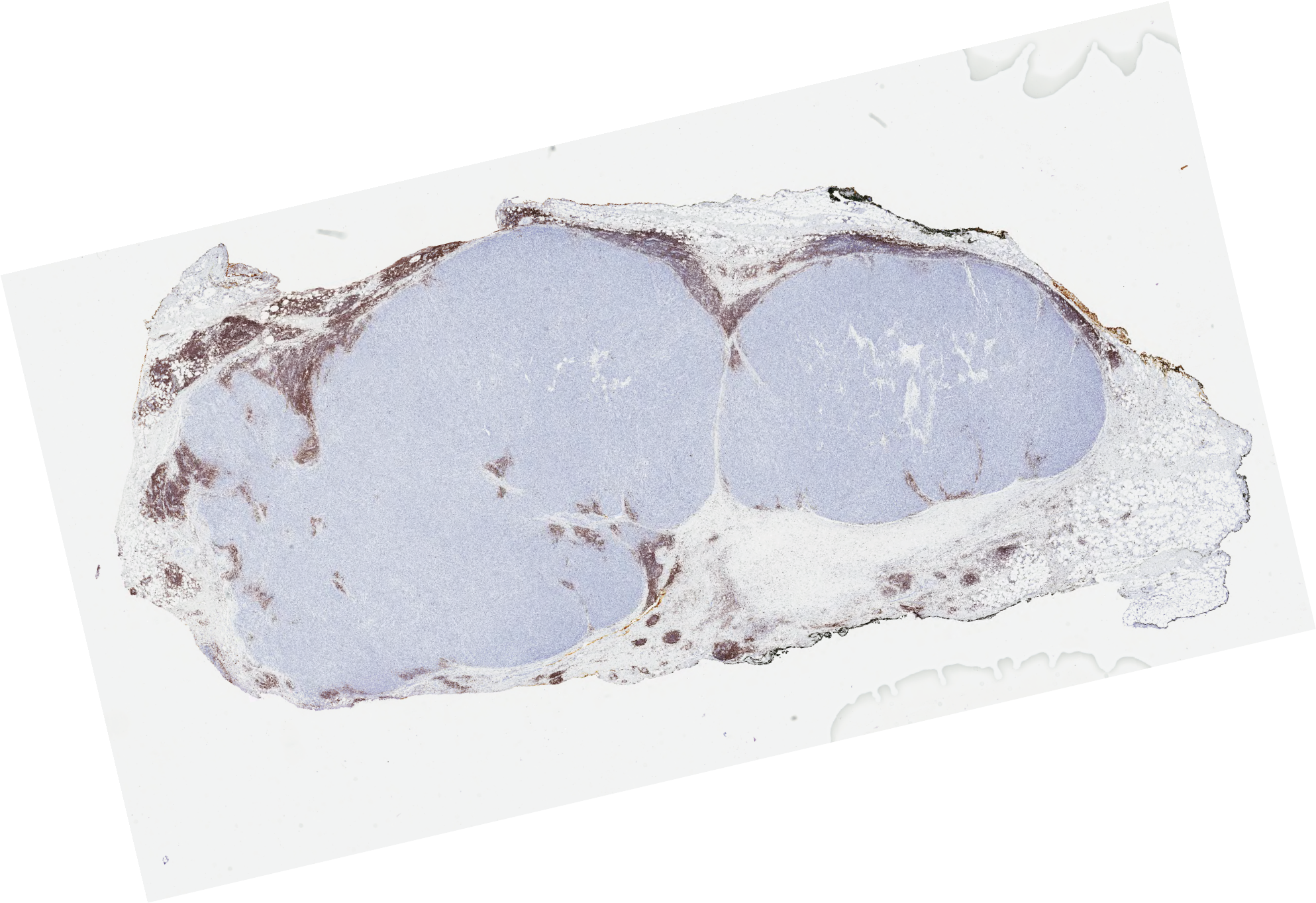

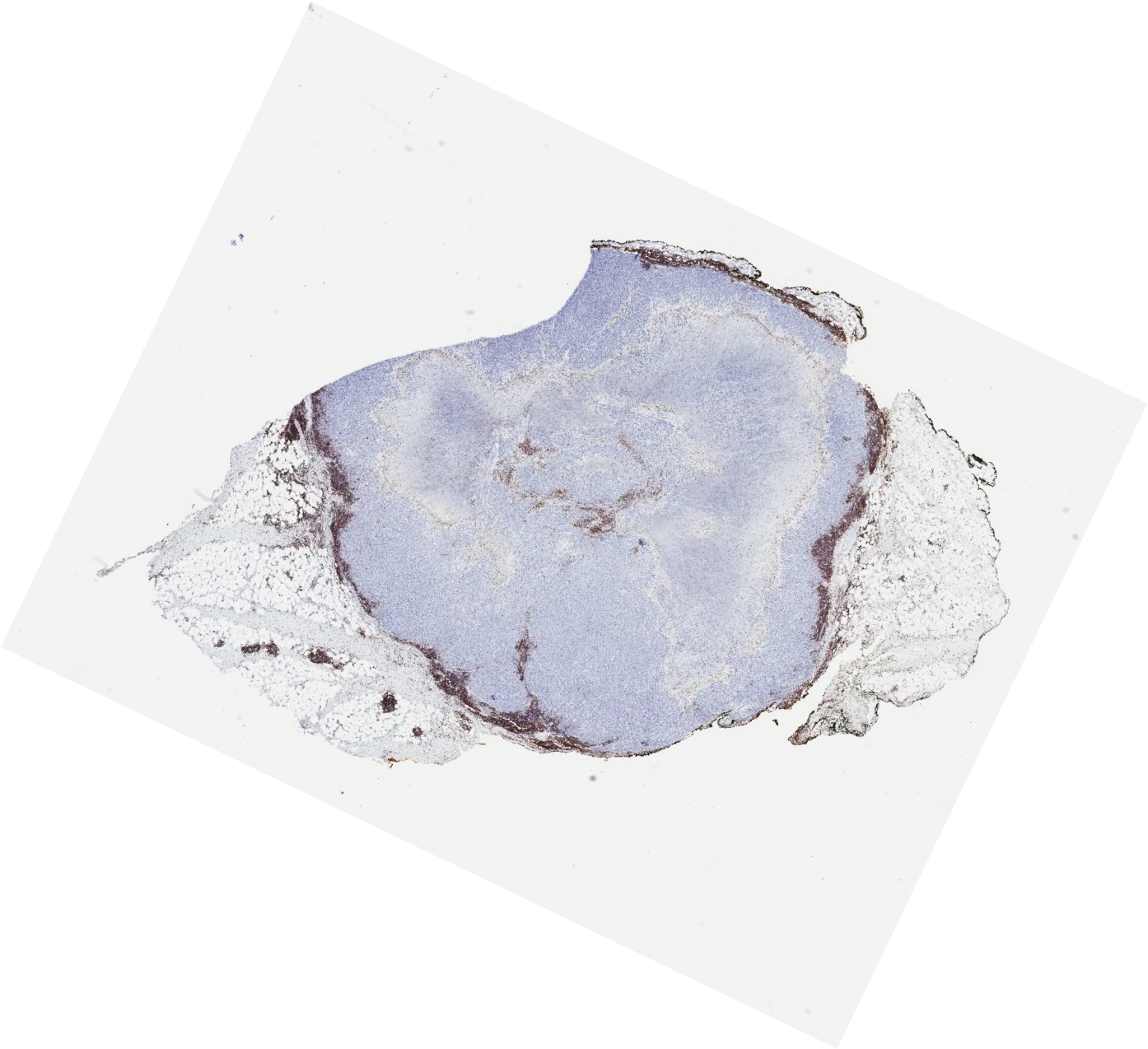

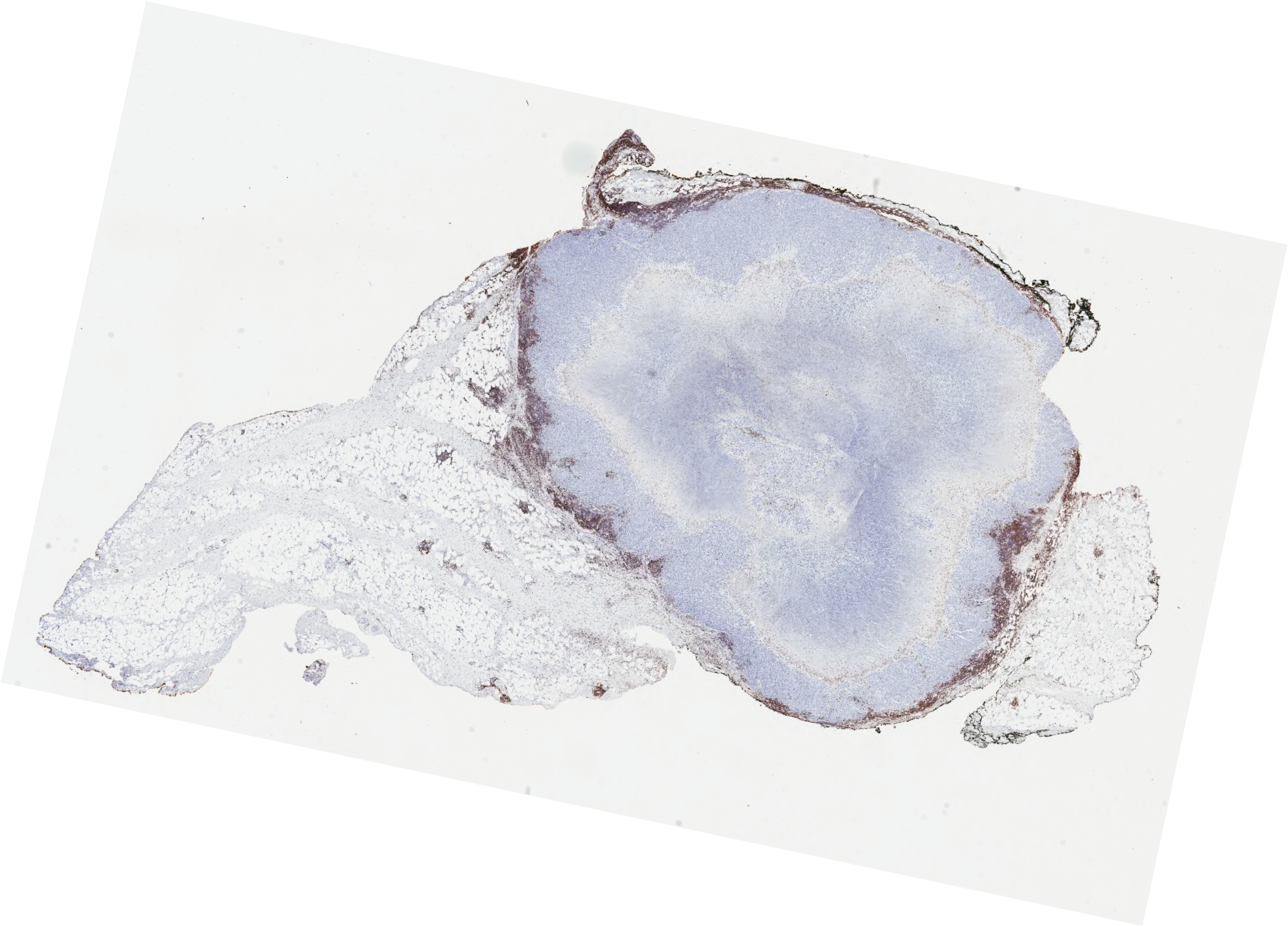

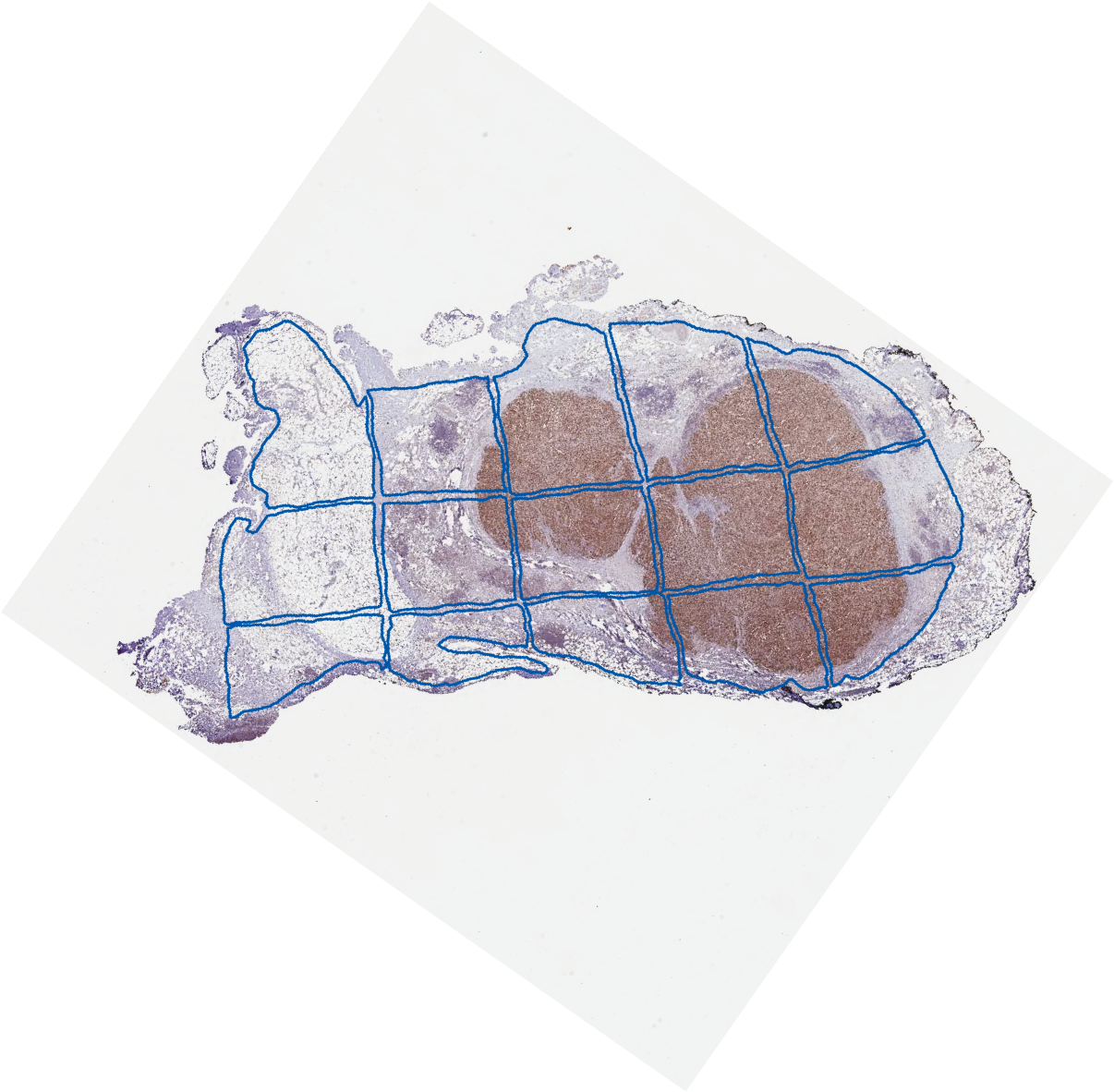

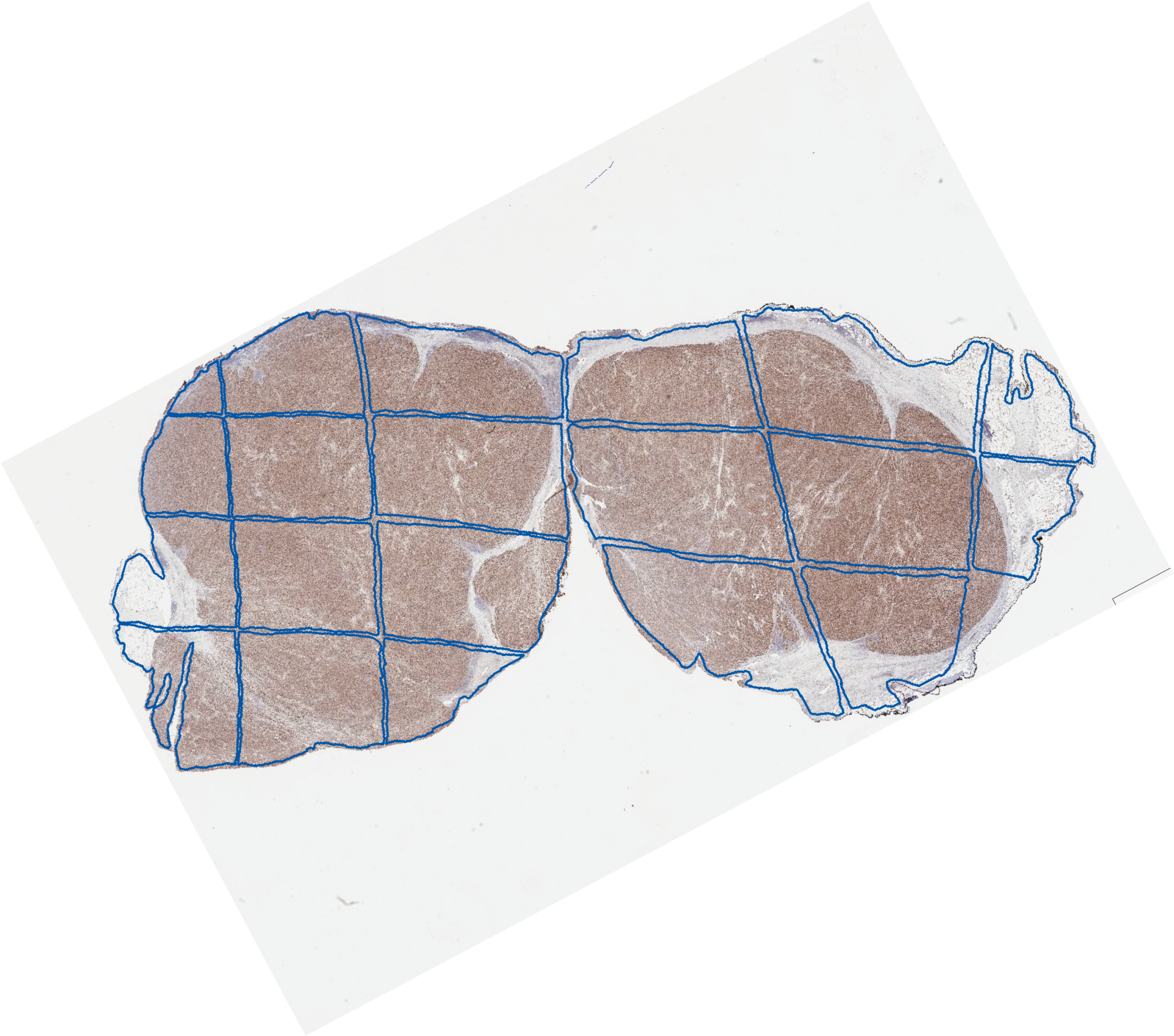

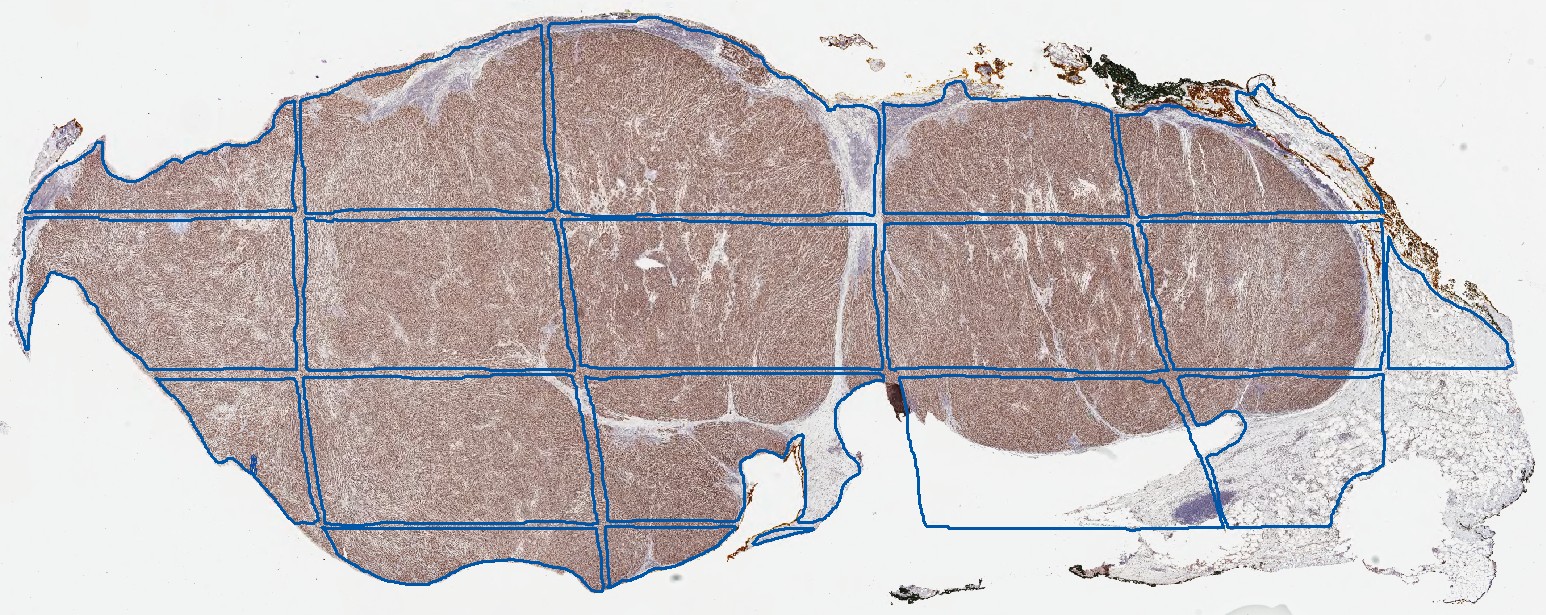

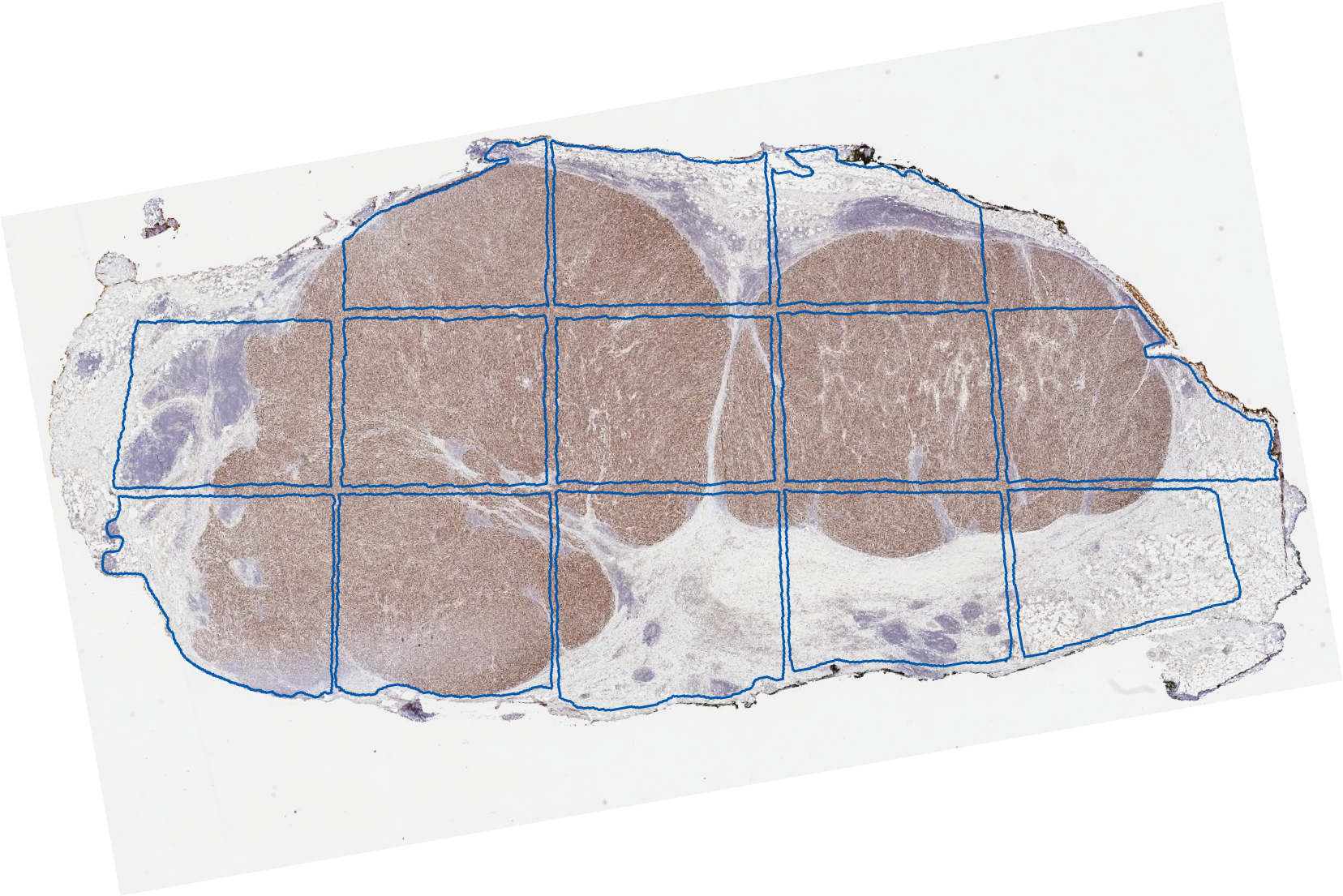

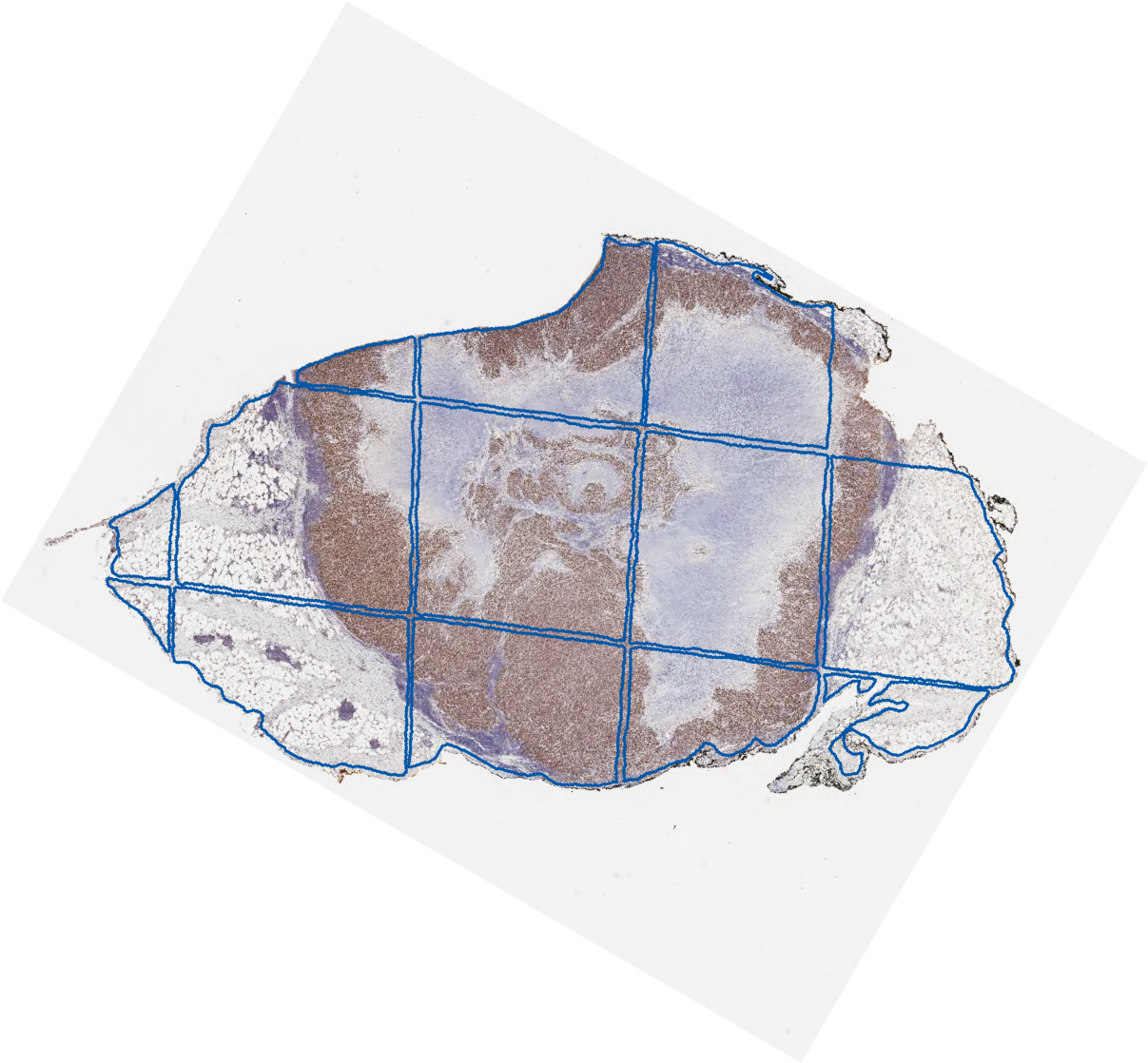

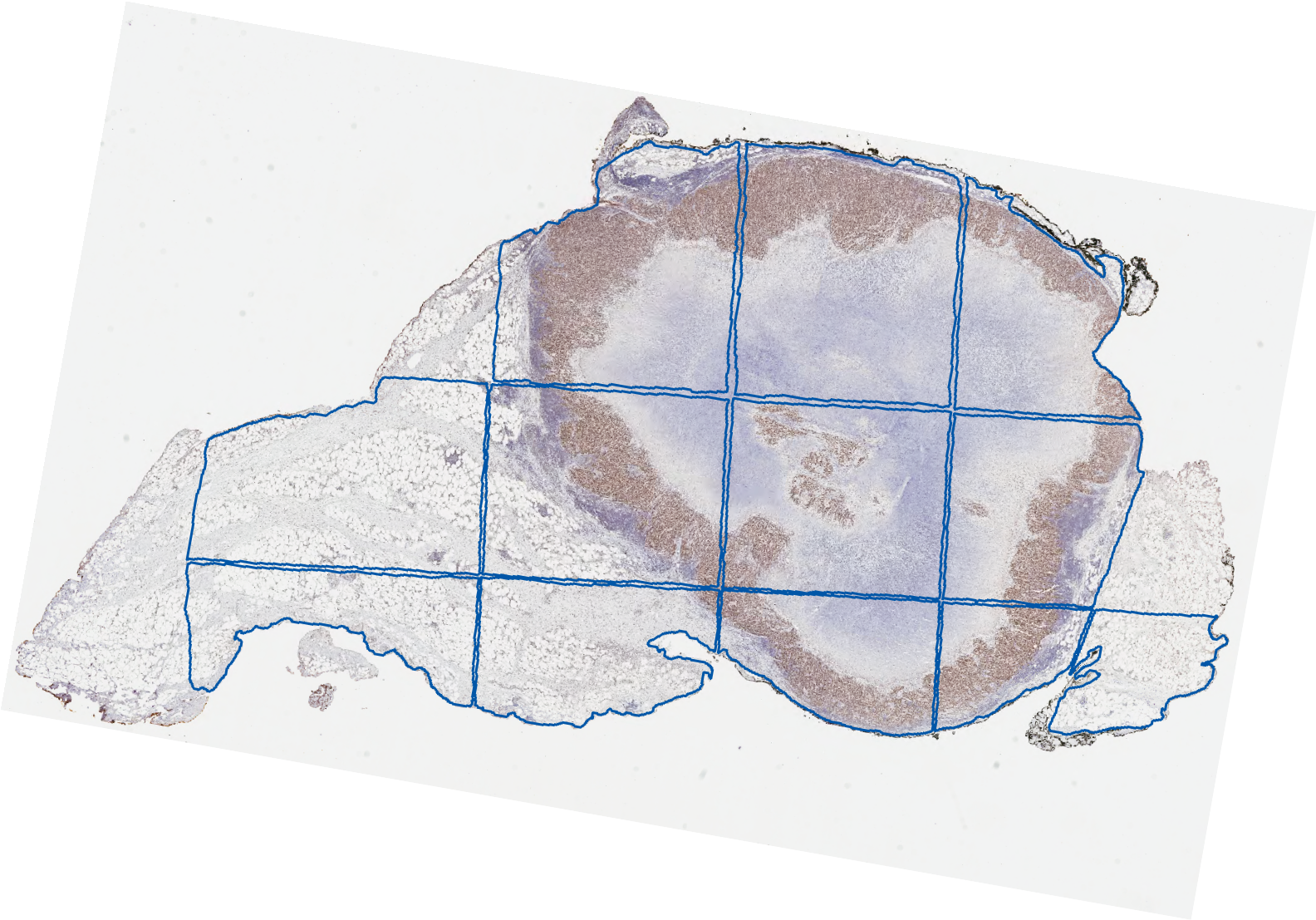

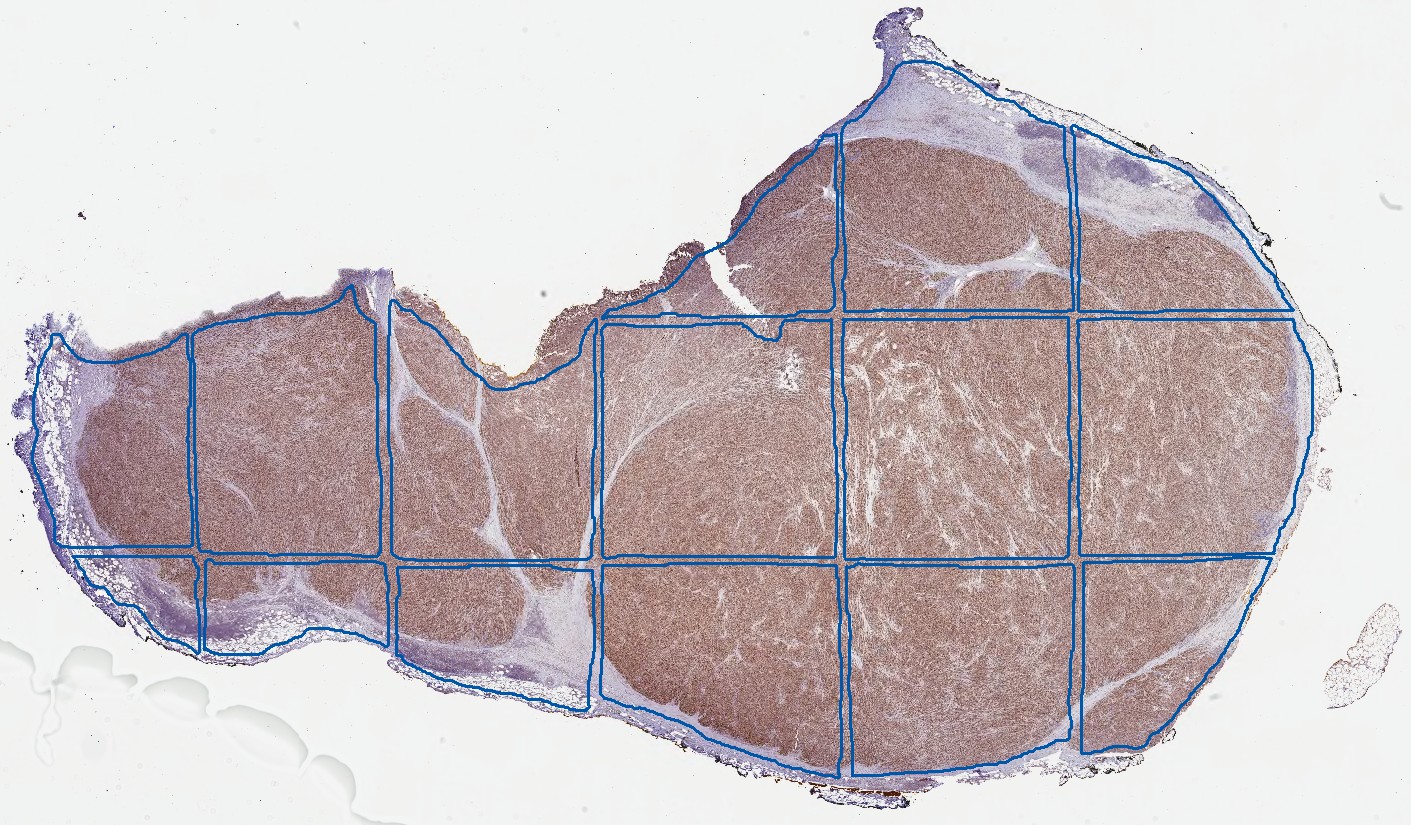


**SOX10 CD45 LCA**

**Frozen Section 2A**

**Section 3A**

**Section 3B**

**Frozen Section 4A**

**Section 5A**

**Section 5B**

**Frozen Section 6A**

**Section 7A**

**Section 7B**

**Frozen Section 8A**

**Supplementary Figure 1: Matched immunohistochemistry sections were used to infer tumor and immune composition of genomic sections.** Shown are IHC stains for tumor (SOX10) and leukocytes (CD45LCA) from FFPE (odd-numbered) tumor sections, providing information from above and below each intercalating frozen (even-numbered) tumor section in order to infer tumor microenvironment composition based on multiple IHC stains. Sub-division of sections into sub-regions in order to study intratumoral heterogeneity was performed on a section- by-section basis on frozen sections, and analogous boundaries applied to adjacent FFPE sections as indicated by blue lines on the SOX10-stained sections.

3

# Supplementary Figure 2.

**A B**

8A8

8A3

8A2

8A12

6A9

6A5

6A3

6A2

6A17

6A16

6A15

6A11

6A10

4A8

4A7

4A4

4A3

4A20

4A2

4A19

4A18

4A17

4A16

4A14

4A13

4A12

4A11

4A10

2A9

2A8

2A7

2A6

2A3

2A2

2A16

2A15

2A14

2A13

2A12

2A10

2A1

Treatment-naive

Post-PD-1i

2 4 6 8 10 12 14 16 18 20 22 24


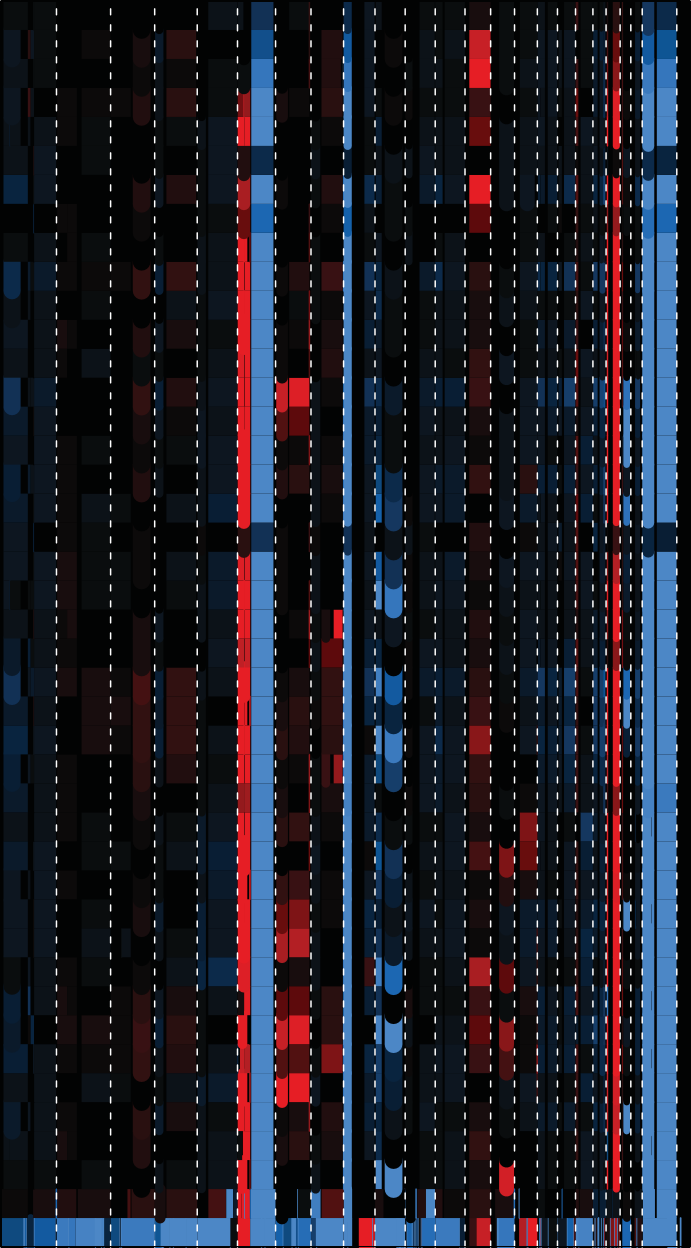


1 3 5 7 9 11 13 15 17 19 21 23


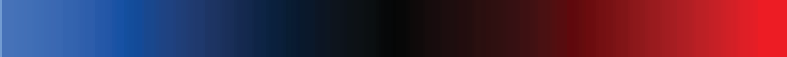


-0.3 -0.15 0 0.15 0.3

copy number (log2(R))

0.5

0.0

Log R

−0.5

0.5

0.0

Log R

−0.5

0.5

0.0

Log R

−0.5

0.5

0.0

Log R

−0.5

0.5

0.0

Log R

−0.5

**Treatment-naive**


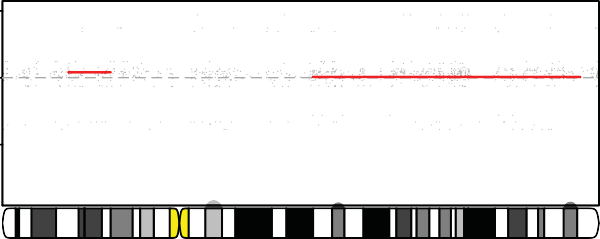


**On PD-1 inhibitor: region 2A1**


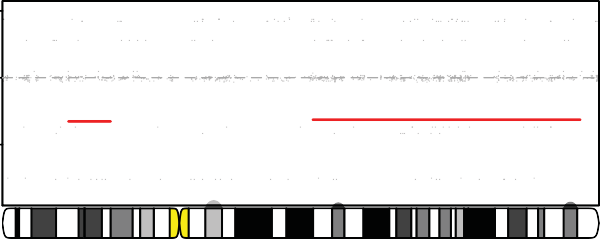


**On PD-1 inhibitor: region 2A16**


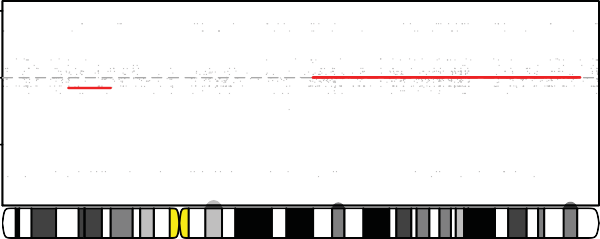


**On PD-1 inhibitor: region 4A12**


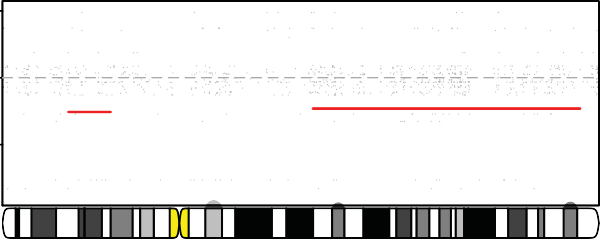


**

* * *

*

*

* * * * * ***

*

*

****

**Post PD-1 inhibitor**


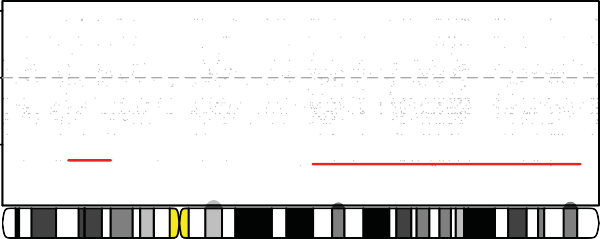


* * * * * * * ** * * ** * * * *

* * * **

* * ** * * ** * *

**Supplementary Figure 2: Inter-tumoral analyses reveal longitudinal copy number evolution.** (**A**) Copy number changes across the on-PD-1 inhibitor lesion demonstrate notable intra-tumor heterogeneity, and additional longitudinal/inter-tumoral heterogeneity in comparison to the treatment-naïve and post-PD-1 inhibitor lesions. (**B**) Chromosome 10 copy number alterations spanning metachronous tumors indicate step-wise losses (indicated by red bar, relative to a zero baseline) in tumors sampled at treatment-naïve, on-PD-1 inhibitor (regions 2A1, 2A16, 4A12, demonstrating ITH) and post-PD-1 inhibitor time points. Data are log2(R) where R is the probe intensity expressed as (observed intensity/reference intensity).

5

# Supplementary Figure 3.

**A B**


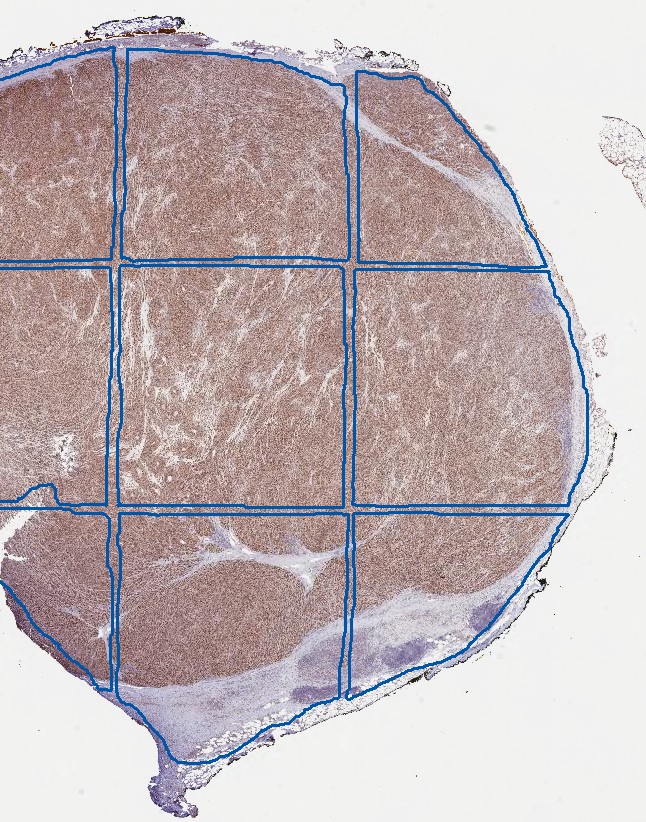


core

high- purity margin

medium- purity margin


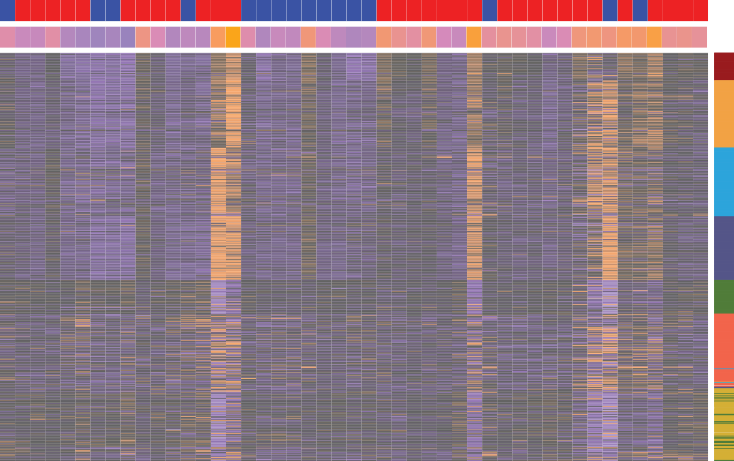
**Immune infiltrate (IHC) Immune score (ESTIMATE)**

**ESTIMATE score and Row z-score**


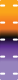
10

5

0

-5

-10

**Cell Type**


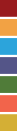
T cells Macrophages Endothelial cells CAFs

Melanocytic AXL-related MITF-related

**Location**


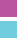
 core

margin


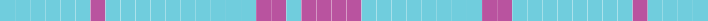

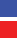
**Immune infiltrate** focal/low moderate/high

2A1

2A2

2A3

2A4

2A6

2A7

2A8

2A9

2A10

2A12

2A13

2A14

2A15

2A16

4A1

4A2

4A3

4A4

4A7

4A8

4A10

4A11

4A12

4A13

4A14

4A16

4A17

4A18

4A19

4A20

6A3

6A5

6A9

6A10

6A11

6A15

6A16

6A17

8A2

8A3

8A4

8A6

8A7

8A8

8A10

8A11

8A12

# C


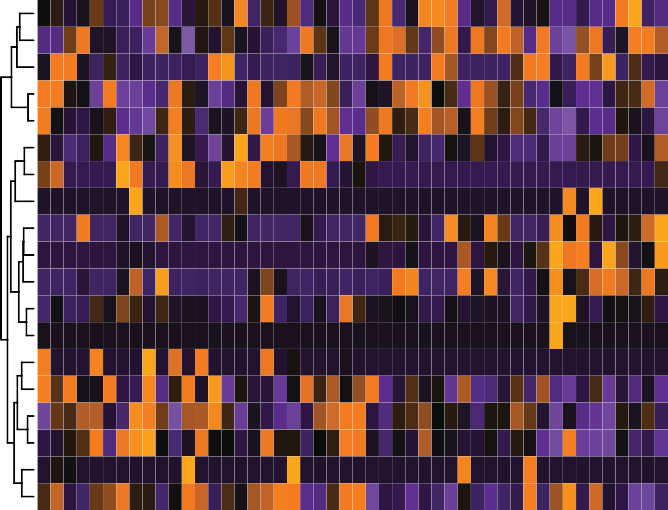
Dendritic.cells.resting


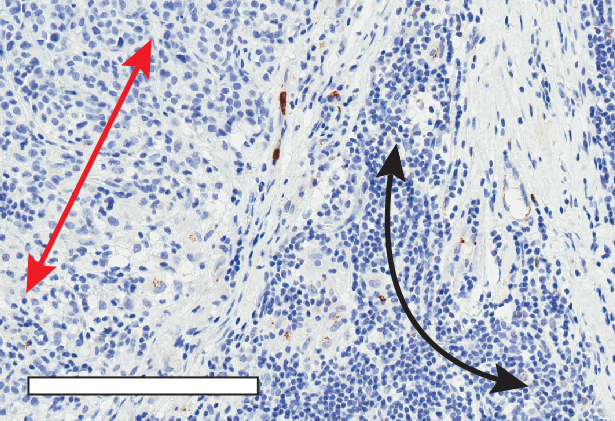


**Tumor**

**Peri-tumoral Leukocytic infiltrate**

200μm

**D Section 5A**

**Sample location**

T.cells.CD4.memory.resting B.cells.naive

Eosinophils Macrophages.M1

T.cells.CD4.memory.activated T.cells.CD8

Neutrophils B.cells.memory T.cells.CD4.naive Dendritic.cells.activated Monocytes NK.cells.resting Plasma.cells Macrophages.M0 NK.cells.activated Mast.cells.resting T.cells.follicular.helper Macrophages.M2

**Section 7A**


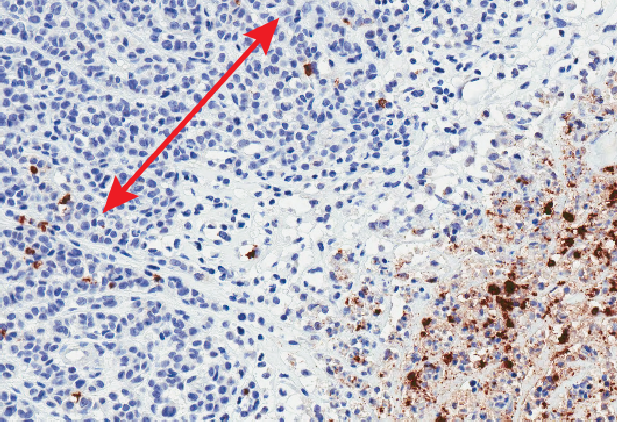


**Tumor**

**Necrosis**

# E

2A2

2A3

2A6

2A7

2A8

2A10

2A12

2A13

2A14

2A16

4A3

4A4

4A7

4A8

4A10

4A11

4A12

4A13

4A14

4A16

4A17

4A18

4A19

4A20

6A3

6A5

6A9

6A10

6A11

6A16

8A8


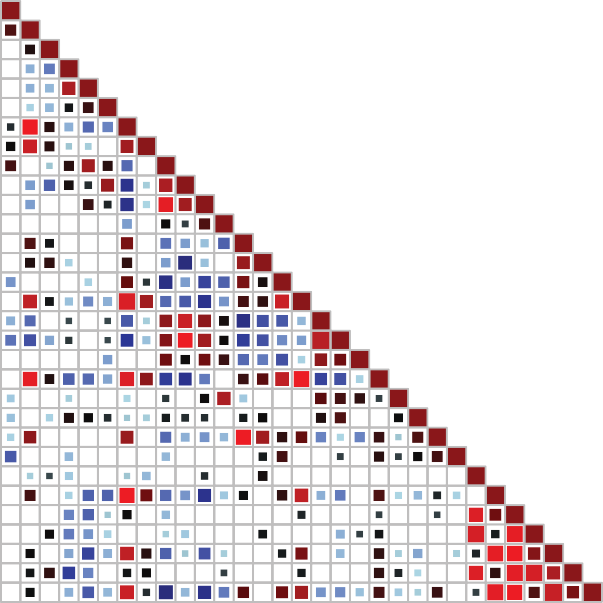
2A2

2A3

2A6

2A7

2A8

2A10

2A12

2A13

2A14

2A16

4A3

4A4

4A7

4A8

4A10

4A11

4A12

4A13

4A14

4A16

4A17

4A18

4A19

4A20

6A3

6A5

6A9

6A10

6A11

6A16

8A8


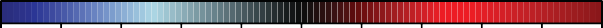


**normalized CIBERSORT relative abundance**


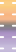
4

2A1

2A2

2A3

2A4

2A6

2A7

2A8

2A9

2A10

2A12

2A13

2A14

2A15

2A16

4A1

4A2

4A3

4A4

4A7

4A8

4A10

4A11

4A12

4A13

4A14

4A16

4A17

4A18

4A19

4A20

6A3

6A5

6A9

6A10

6A11

6A15

6A16

6A17

8A2

8A3

8A4

8A6

8A7

8A8

8A10

8A11

8A12

8A13

2

0

−2

−4

#
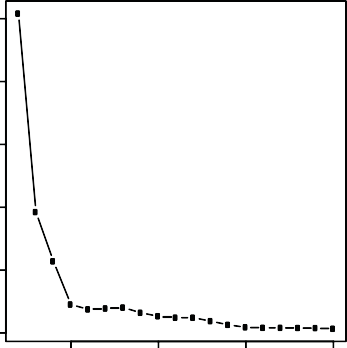
F

0.5

relative change in area under CDF curve

0.4

0.3

0.2

0.1

0

Delta area

2

3

4

5

6

7

8

9

k 10

11

12

13

14

15

16

17

18


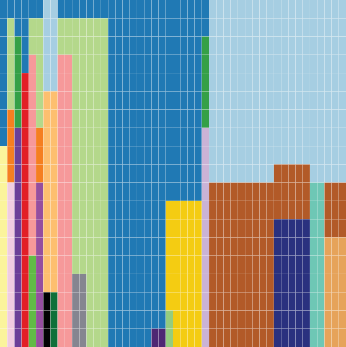
19

Tracking plot

−1 −0.8 −0.6 −0.4 −0.2 0 0.2 0.4 0.6 0.8 1

Spearman correlation

5 10 15 20

k (number of clusters)

20

Samples

**Supplementary Figure 3: Deconvolution of melanoma and immune cell transcriptomic and proteomic signatures.** (**A**) Example histologic appearances (SOX10 stain) of subregions designated as core, high-purity margin and medium-purity margin. (**B**) Supervised clustering (in spatial order) of the transcriptomic profile of samples derived from the on-PD-1 inhibitor tumor lesion, highlighting tumor-derived and immune cell subset-derived signatures. IHC-based immune infiltrate and ESTIMATE immune scores (top) and IHC-based sample location (bottom) are indicated. (**C**) Heatmap of immune infiltrate composition deconvoluted from transcriptomic data using CIBERSORT. (**D**) Example of low-density (top) and high-density (bottom) CD15+ staining as a marker for the presence of neutrophils. The majority of CD15 staining was observed within necrotic regions of FFPE slice 7. (**E**) Sample inter-correlation matrix based on reverse-phase protein array quantification of protein expression, illustrating dispersed protein expression patterns throughout sub-regions of the on-PD-1 inhibitor tumor. (**F**) Delta area (left) and tracking plot (right) of consensus clustering of samples using transcriptome data to identifying the optimal number of sample clusters that captures the majority of intra-tumoral heterogeneity signatures. A total of 4 clusters provided the greatest balance between information and similar cluster size, whilst >4 cluster-solutions contributed lower-yield clusters containing few samples each.

7

# Supplementary Figure 4.

**A B**

T cell receptor signaling pathway


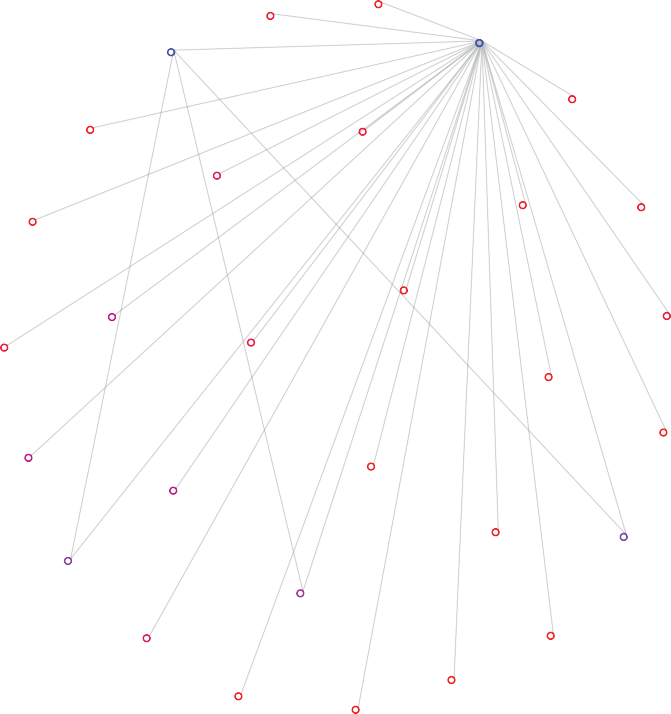


IKZF1

HHEX

●

FLT3


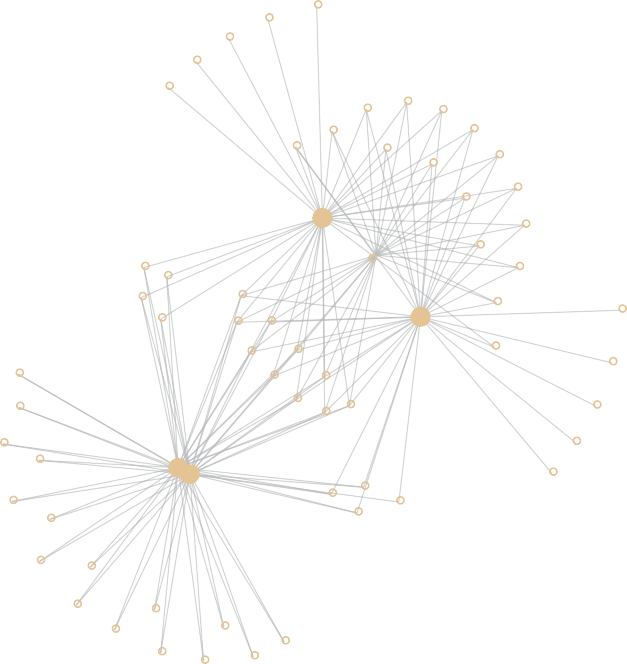
●

B cell proliferation

●

positive regulation of cell adhesion

●

regulation of leukocyte proliferation

●

IKZF3●

●

● ●

T cell selection

MFNG

TNFSF8

CCL19

●

- ● ●RHOH

immune response−activating cell surface receptor signaling pathway

CD1D

T●CF7 ● ●

JAK3●

- SLAMF6

IL7R

regulation of immune effector process

●

lymphocyte differentiation

GPR183

●

●

●

BCL11B

●

●

regulation of leukocyte mediated immunity

mononuclear cell proliferation

● ●

SASH3

RO●RC

●

immune response−regulating cell surface receptor signaling pathway

positive regulation of T cell activation

CR2 PLCG2

●

T cell diff●erentiation

● IL23R

●IL6

regulation of antigen receptor−mediated signaling pathway

●

FCRL3●

LCK

●

RASGRP1

●

NLRC3

●

regulation of lymphocyte proliferation ●

CD79A ●

ZAP70 ●

CD3G

●

T cell activation

● ●

positive regulation of cell activation ●

leukocyte cell−cell adhesion

PRKCB

●

MUC3A

●

UBASH3A

CD3D● TESPA1●

immune response−activating cell surface receptor

CD3E

●

CR1

●

- CD28 ●PTPRC ITK

●

● CD40LG

ICOS

●

TREML2

●

●

- - positive regulation of leukocyte cell−cell adhesion

positi ve regulation of cell−cell adhesion

lymphocyte proliferation

●

T cell differe ntiation

●

- 1. djust

2e−07


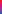


4e−07

6e−07

●

BLK●

ELMO1

signaling pathway

immune response−regulating

● ●CD300A

CLECL1

● CD1C

- - - lymphocyte differentiation

●

regulation of mononuclear cell proliferation

●

8e−07

- - - - ● cell surface receptor GRAP2 ●
- BTLA

B cell activation

TRAT1

●

RFTN1

signaling pathway

PRKCQ

regulat ion of lymphocyte mediated immunity

●

● ●

positive T cell selection

TRAC ● STAP1●

●

- CD226

CLEC10A

●

regulation of B cell receptor signaling pathway

●

positive regulation of leukocyte activation

●

●

FGR

● CD22 ●

antigen receptor−mediated signaling pathway

CD247 ●

● PAX5

ICAM2

leukocyte proliferation

●

●

●

T cell activation

alpha−beta T cell activation

**Supplementary Figure 4: Transcriptional heterogeneity at margin sites.** (**A**) Gene connection network of differentially-expressed genes between samples located at the tumor margin comparing those with high versus low immune infiltrates. (**B**) Functional annotation network of differentially-expressed genes between high versus low immune infiltrate tumor margin samples, showing enrichment for T and B lymphocyte pathways and functions.

9

# Supplementary Figure 5.

15


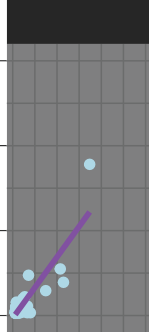


2A1

CSVPTSGSRDNEQFF

CASSLHGDQPQHF

CASSNTGGSLDGYTF CASSLVREGEQYF


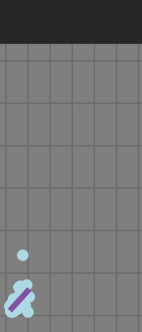


2A10

CASSLGGQGGETQYF

CSVQPTGSQGRSGRTDENAEFQFFF CASSLHGDQPQHF


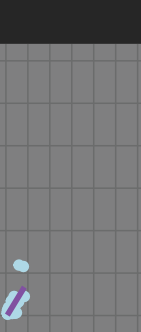


2A12

CAVSPSTSSLSGGSARIDLNEQFF

CASSLEVDRRESGYENQEYQFFF


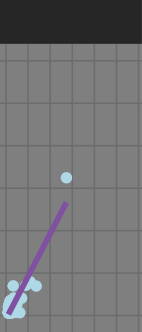


2A14

CSVPTSGSRDNEQFF

CCAASSSLHQLVGRDAERGRPEEQEHYTFFQYF


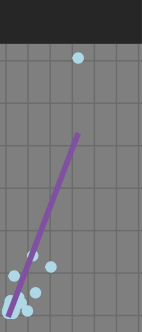


2A15

CSVPTSGSRDNEQFF

CASSLQGARREETQYF CASSLHGDQPQHF

CASSLVREGEQYF


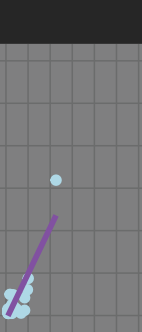


2A16

CSVPTSGSRDNEQFF

CASSLQGARREETQYF

CASSLHGDQPQHF CASSLVREGEQYF


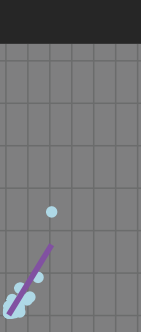


2A2

CSVPTSGSRDNEQFF

CASSLHGDQPQHF CASSSLSGAILNEQFF CASSLVREGEQYF


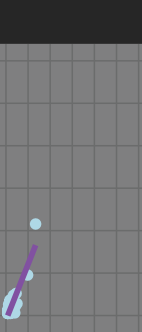


2A3

CSVPTSGSRDNEQFF

CASSLHGDQPQHF

CASSLSQLSGGAARIRLNEETQQFYFF


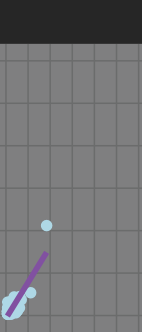


2A4

CSVPTSGSRDNEQFF

CASSLHQGDAQRRPQEEHTFQYF

CASSEDRSYNEQFF


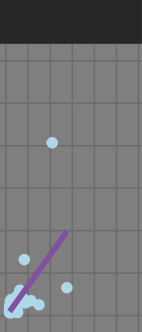


2A7

CSVPTSGSRDNEQFF

CASSLGGQGGETQYF

CASSLSAPISGGATYEQYF CASSLHGDQPQHF


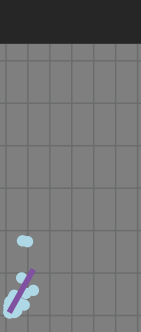


2A8

CAVSPSTLGSGQRGDGNEETQQFYFF

CSVQGQRGTEAFF CASSLSAPISGGATYEQYF


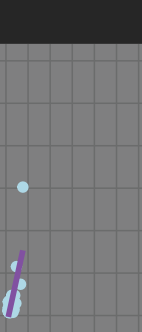


2A9

CSVPTSGSRDNEQFF

CASSLGGQGGETQYF

CASSLHGDQPQHF CASSSLSGAILNEQFF

10

5

0

15


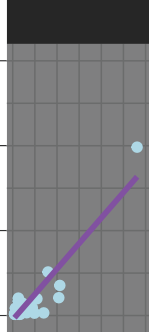


4A1

CSVPTSGSRDNEQFF

CASSLQGARREETQYF CASSLHGDQPQHF CASSLVREGEQYF


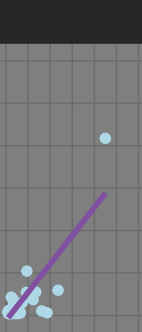


4A10

CSVPTSGSRDNEQFF

CASSLQGARREETQYF

CASSCLAHSGSDLQVRPQEGHFEQYF


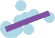


4A11

CASTPGGLVFNEQFF CASCSSESVDLPSRTGSAYGINLSENRQEDQFNFFEFQFF


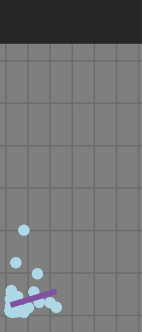


4A13

CSVPTSGSRDNEQFF

CSVQGQRGTEAFF

CASSFGSRVGETQYF CASSLVREGEQYF


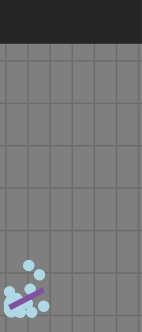


4A14

CSVQGQRGTEAFF CSVPTSGSRDNEQFF

CASSLHGDQPQHF

CASSLVREGEQYF


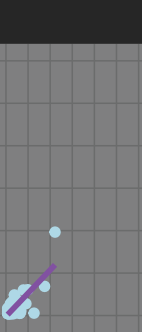


4A16

CSVPTSGSRDNEQFF

CASCSANSLTSSGLGVGRASIELGNEGEQYYTFF


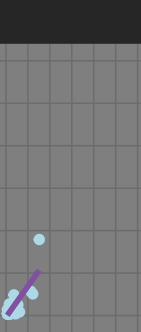


4A17

CSVPTSGSRDNEQFF

CASSSSNLQVTRGEGAGRSERLQDEGYEFTYQTFYF


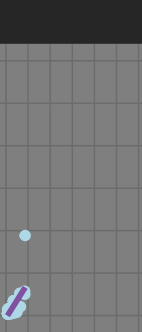


4A18

CSVPTSGSRDNEQFF

CASSLSVHLRSGEGDGAQEIPLQNQYEHFQFFF


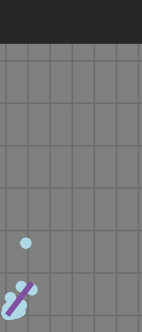


4A19

CSVPTSGSRDNEQFF

CCAASSSSSLLHLVSGRGDEAGQIEPLNQEHQFFF


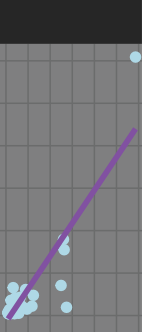


4A2

CSVPTSGSRDNEQFF

CASSLQGARREETQYF CASSLHGDQPQHF

CASSLVREGEQYF


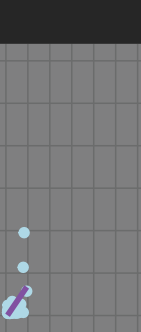


4A20

CSVPTSGSRDNEQFF

CASSSLSGAILNEQFF

CASSLHGDQPQHF CASSNTGGSLDGYTF


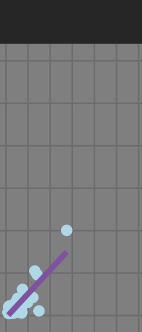


4A3

CSVPTSGSRDNEQFF

CCAASSSSSLLHSGGDAQILPNQEHQFFF

CASSEDRSYNEQFF

10

Productive frequency of RNA clones (%)

5

0

15


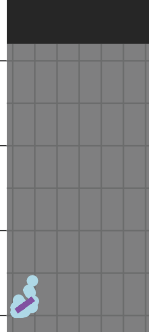


4A4

CSVPTSGSRDNEQFF CSAVSQSLGGQGRQGGTGEAEFTFQYF CASSSLSGAILNEQFF


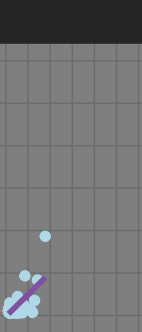


4A7

CSVPTSGSRDNEQFF

CASSLSHLSGGDAQIPLNQEHQFFF

CASSEDRSYNEQFF


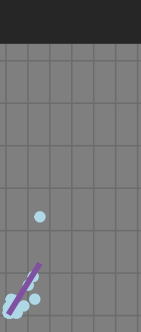


4A8

CSVPTSGSRDNEQFF

CASSLHGDQPQHF CASSSLSGAILNEQFF

CASSLVREGEQYF


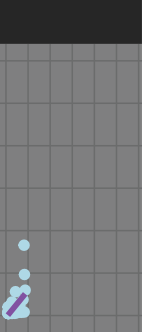


6A10

CSVPTSGSRDNEQFF

CASSSLSGAILNEQFF

CASSLHGDQPQHF CASSEDRSYNEQFF


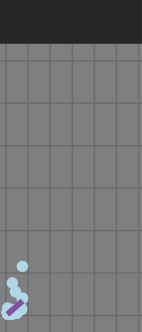


6A11

CASSSLSGAILNEQFF

CASTPGGLVFNEQFF

CASSELHDGRDSYQNPEQQHFF


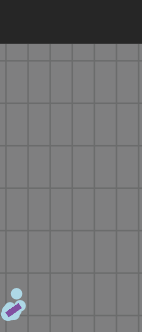


6A13

CSVPTSGSRDNEQFF CASSLLHQDGRDAQRNRPEQQEHTFQF YF


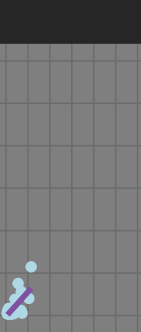


6A16

CASSSLSGAILNEQFF

CSVPTSGSRDNEQFF

CASSLHGDQPQHF

CASSEDRSYNEQFF


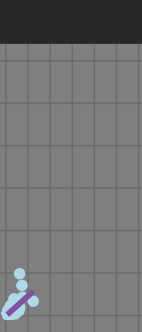


6A2

CSVPTSGSRDNEQFF CASSSLSGAILNEQFF CASSLEHDGRDSQYNPEQQHFF


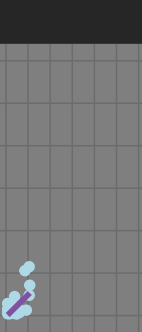


6A3

CASSVSPSTSLSGGSARIDLNEQFF

CASSLHGDQPQHF CASSEDRSYNEQFF


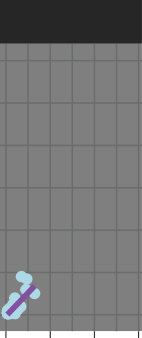


6A4

CAVSPSTSSLSGGSARIDLNEQFF CASSLEHDGRDSQYNPQEQHFF


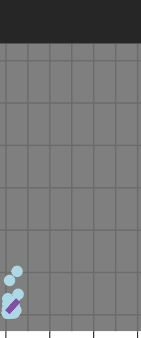


6A5

CSVPTSGSRDNEQFF CASSSLSGAILNEQFF

CASSLHGDQPQHF CASSEDRSYNEQFF


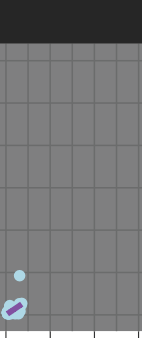


6A9

CSVPTSGSRDNEQFF

CASSELHDLSGRGDSAYQNIPLENQQEHQFFF

10

5

0

0 2 4 6 0 2 4 6 0 2 4 6


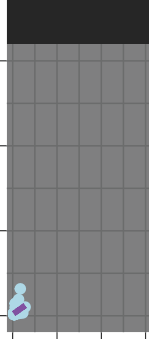


8A10

CASSSLSGAILNEQFF

CASTSPLHGGLVQFPNQEHQFFF

CASSEDRSYNEQFF


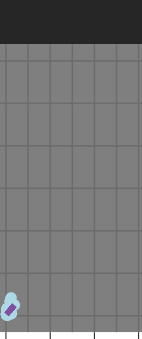


8A11

CASSTPSGLSGGLVAFILNNEEQQFFFF

CASSEDRSYNEQFF

CASRPGFGNTEAFF


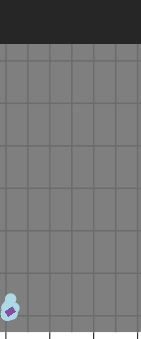


8A12

CASSTPSGLSGGLVAFILNNEEQQFFFF

CASSVEGDRKGSYGNQEVQDFTFEAFF


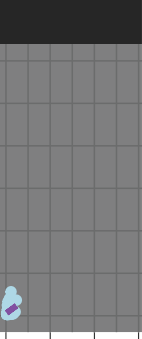


8A2

CASSSLSGAILNEQFF

CASSTPEGDRGSLVYFNNEEQQFFFF CASSLVEGMEQYF


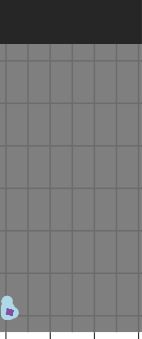


8A3

CSVPTSGSRDNEQFF

CASSVESGLSRKGGSAYGINLQNVQEDQFTFEFAFF


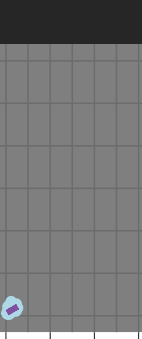


8A4

CAVSPSTELSDGRRSPYDNNEEQEQFYF F

SAGSTGGPTYEAQFFF


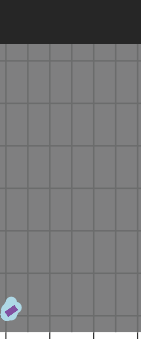


8A6

CASSTSPLESGDLSGRGSLVAYFINLNENEQEQQFFFFF

VEGMEQY


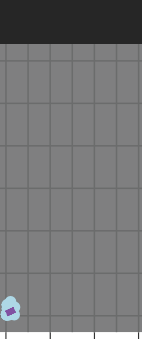


8A7

CASSTSPESGDLSGRGSLVAYFINLNENEQEQQFFFFFF

VGKGGQVDTEAFF


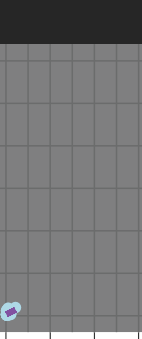


8A8

CASSVESGLSRKGGSAYGINLQNVQEDQFTFEFAFF

CASSLVEGMEQYF

15

10

5

0

0 2 4 6 0 2 4 6 0 2 4 6 0 2 4 6 0 2 4 6 0 2 4 6 0 2 4 6 0 2 4 6 0 2 4 6

Productive frequency of DNA clones (%)

**Supplementary Figure 5: Identification and distribution of dominant TCR clonotypes.** TCR clonal activation inferred from matched TCR-Vβ DNA and RNA sequencing identifies dominant clonotypes and their spatial distribution. Data represent productive frequencies within the total TCR repertoire derived from either DNA-based or RNA-based sequencing, expressed as percentages.

11

# Supplementary Figure 6.

**TCR clonotype (amino acid sequence)**

CASSLSAPISGGATYEQYF

CASSLQGARREETQYF

CASSTSNGLAGVNEQYF

CASSLHGDQPQHF

CASSNTGGSLDGYTF

CASSESEGDTF

CASASSNSYEQYF

CASSLVREGEQYF

CASSILRELSEQYF

**Gene harboring mutation**

CSVPTSGSRDNEQFF

ADAM7

AIFM3

BRAF

CDC42EP4

CDKN2A

GAGE1

IFNA7

JAK1

KMT2A

MAP2K1

NCOR1

NRAS

PTPRB

ROBO2

TOP1

**Spearman correlation**

0.61


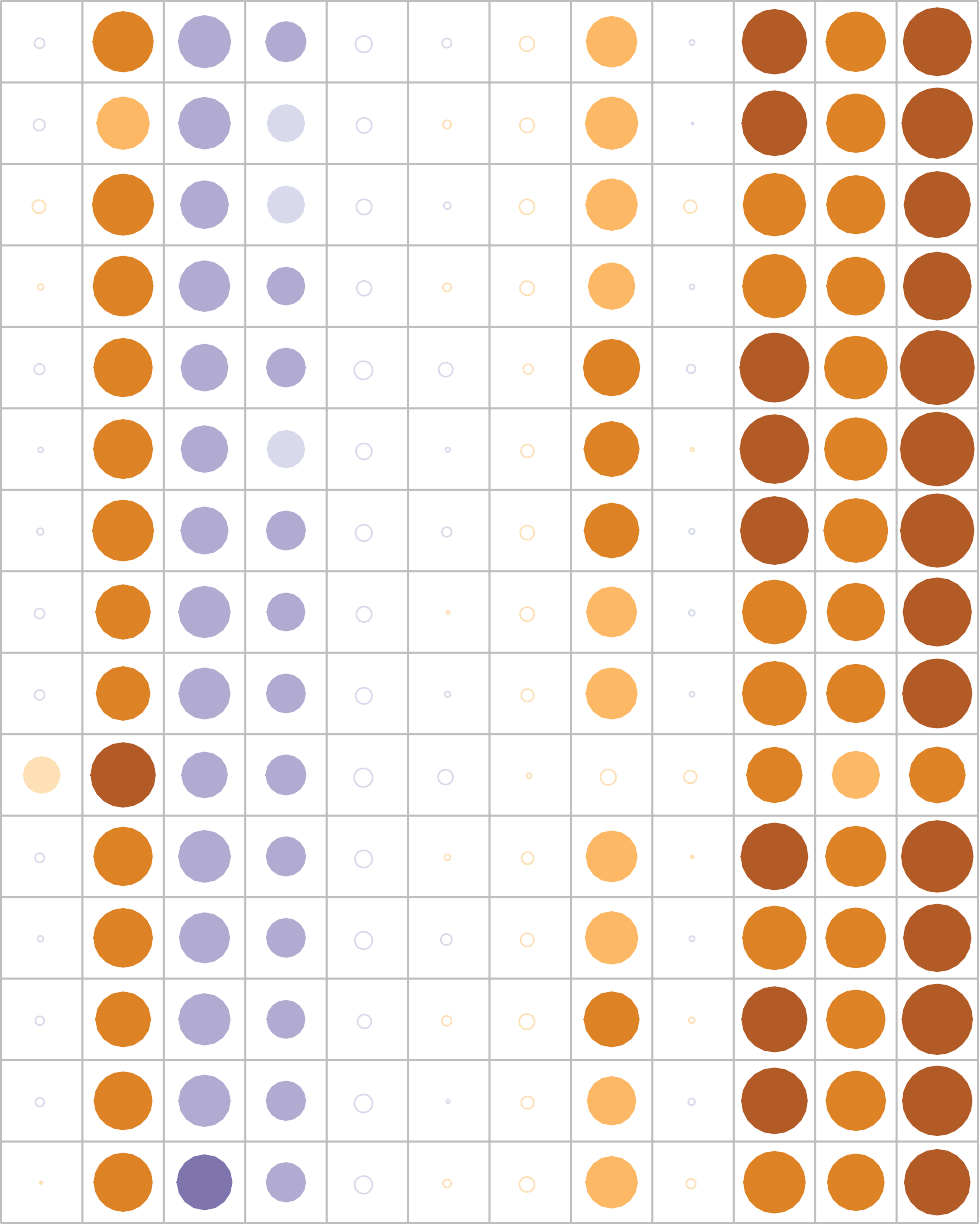


●

●

●

●

●

●

●

●

●

●

●

●

●

●

●

●

●

●

●

●

●

●

●

●

●

●

●

●

●

●

●

●

●

●

●

●

●

●

●

●

●

●

●

●

●

●

●

●

●

●

●

●

●

●

●

●

●

●

●

●

●

●

●

●

●

●

●

●

●

●

●

●

●

●

●

CEVQYF

CSVQGQRGTEAFF

0.48

0.34

0.21

0.07

−0.06

−0.2

−0.34

**Supplementary Figure 6: Associations between high-abundance TCR clonotypes and truncal tumor mutations reveal probable distinct tumor-specific and bystander populations.** Correlations between abundance of high-frequency TCR clonotypes (defined at the amino acid level) and a core set of truncal mutations (variant allele frequency) identified throughout all sub-regions of the on-PD-1 inhibitor tumor. The strength and direction of correlation is indicated by color, whilst the size of each circle indicates the statistical significance (larger indicates more highly significant), with all values meeting the criterion of adjusted p-value < 0.05.

13

# Supplementary Figure 7.

**A B**


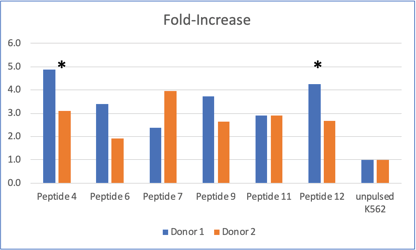


|  | **Donor 1** | **Donor 2** |
| --- | --- | --- |
| **Peptide 4** | **1.9** | **0.74** |
| **Peptide 6** | **1.33** | **0.46** |
| **Peptide 7** | **0.93** | **0.95** |
| **Peptide 9** | **1.45** | **0.63** |
| **Peptide 11** | **1.13** | **0.70** |
| **Peptide 12** | **1.66** | **0.64** |
| **unpulsed K562** | **0.39** | **0.24** |

**C**


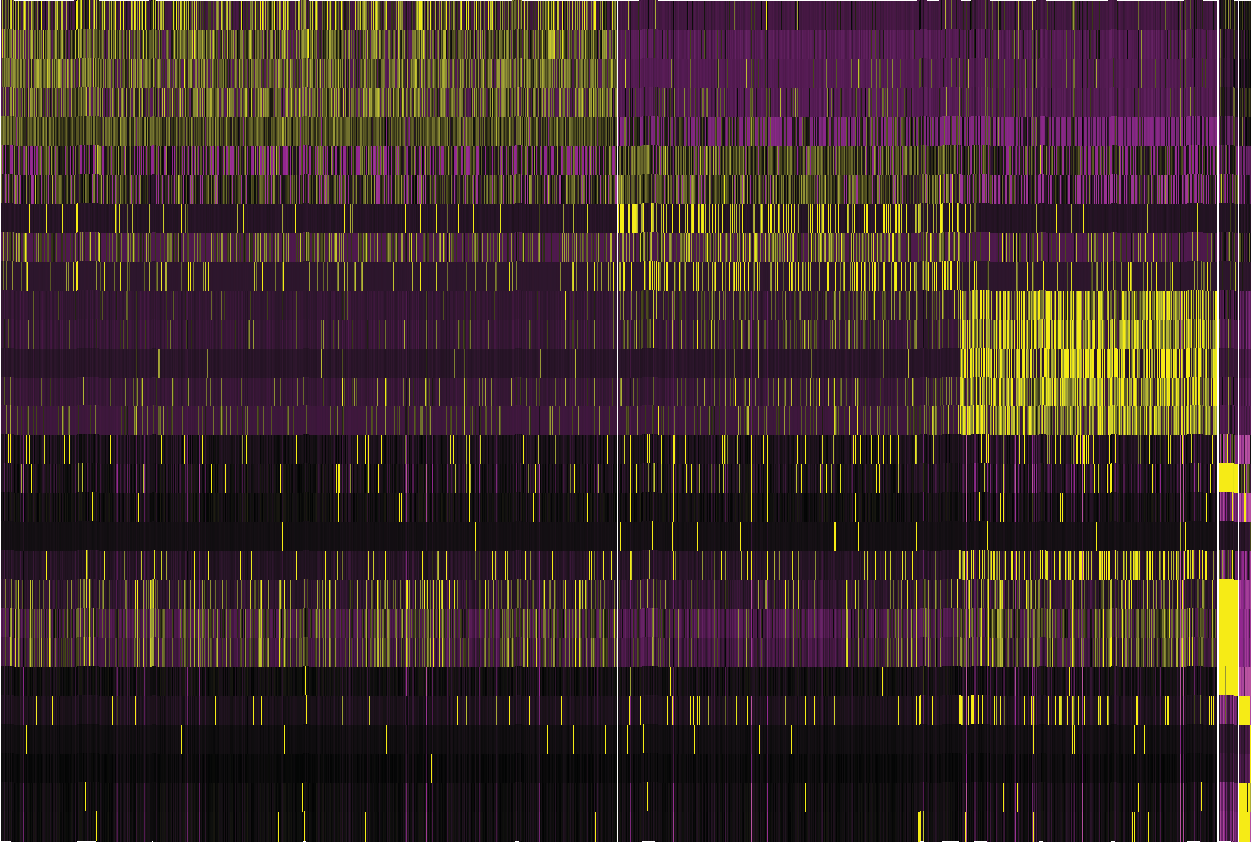


CCL4 NKG7 CD8A GZMK CCL5 IL7R SLC2A3 CD40LG KLF2 KLRB1 TNFRSF4

TNFRSF18 FOXP3 CTLA4 BATF MMP9 CST3 SPP1 CXCL13 ACP5 HLA−DRA HLA−DRB1 HLA−DRB5 LYZ SERPINE2 COL1A1 COL1A2 CRYAB S100A1

Activated CTL

CD4 T cell

Checkpoint-regulated T cell

HLA-rich

ECM remodelling

## Supplementary Figure 7: Single cell sequencing identifies distinct T cell phenotypes. (A-

**B**) Detection of epitope specific CD8+ T cells in the PBMC of HLA-A*0301 healthy donors.

Potential epitope specific CD8+ T cells in PBMC were stimulated three times at 7-day intervals by co-culturing with HLA-A*0301 expressing K562 (A3-K562) pulsed with overlapping peptides (peptides 1-12) in the presence of IL-21. Epitope specificity of sensitized CD8+ T cells was determined by co-culturing cells with peptide pulsed K562 overnight and performing a standard intracellular IFN-γ production assay. Cells co-cultured with non-pulsed K562 cells served as background (negative) controls. Treatment with PMA and ionomycin served as a positive control for the assay. The table (**A**) lists the highest responding peptide candidates as % IFN-γ+ CD8+ T cells among total CD8+ T cells (baseline of 0.39% and 0.24% from Donors 1 and 2, respectively). Fold-increase in peptide reactivity above baseline (baseline = 1.0) is shown in the column chart

(**B**) at right. (**C**) Heatmap of the most differentially-expressed genes between tumor infiltrating lymphocyte clusters defined by tSNE analysis.

15

# Supplementary Figure 8.

**A C**

T−test, p = 3.4e−05 T−test, p = 0.092


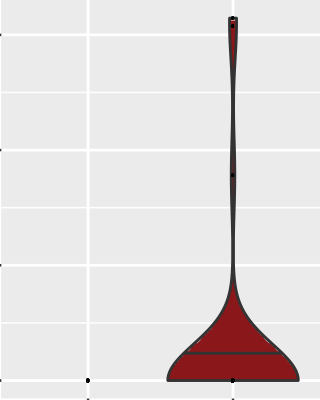


6.0


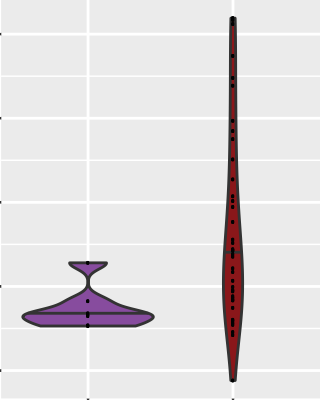


5.5

0.006

Lysoso●me

ECM−receptor interaction

●

Protein digestion an●d absorption

5.0

Neutrophils

4.5

0.004

0.002

Neutrophils

Apoptosis

●

Phagosome

●

Platelet activation

●

Tight junction

●

Amoebiasis

●

- Focal adhesion

●

Vibrio cholerae in●fection ● Leukocyte transendothelial migration

4.0

Bacterial invasion of epithelial●cells

0.000

Legionellosis● ●

Regulation of actin cytoskeleton

Gain Stable

Gain Stable

Dilated cardiomyopathy (DCM)

●

Shigellosis

Chromosome 7 Status

Chromosome 7 Status

Arrhythmogenic right ventricu●lar cardiom●yopathy (ARVC)

● Salmonella infection

● Pathogenic Escherichia coli infection

# B

Adherens junction

●

Proteoglycans in cancer

0.15


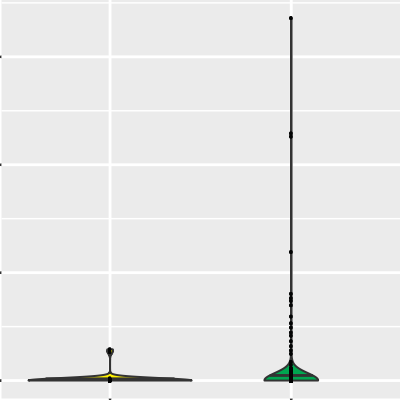


T−test, p = 0.043

Biosynthesis of amino acids

●

●

Fluid shear stress and atherosclerosis

0.10

Neutrophils

Glycolysis / Gluconeogenesis

●

● Necroptosis

Protein processing in endoplasmic reticulum

●

●

0.05

p.adjust

●

HIF−1 signaling pathway

Estrogen signaling pathway

0.00

Gain Stable

0.01

0.02


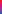


0.03

0.04

●

Antigen processing and presentation

●

Chromosome 7 Status

# D E

T−test, p = 0.49

T−test, p = 0.53

Thyroid hormone synthesis

# F

T−test, p = 0.27

0.009

Neutrophil Score

0.15

0.6

0.006

Neutrophil Score

0.10

0.4

Neutrophil Score

0.003

0.05

0.2

0.000

NR R

Response Status

0.00

NR R

Response Status

0.0

NR R

Response Status

**Supplementary Figure 8: Neutrophil estimation and related Gene Ontology (GO)-term and KEGG pathway enrichments in clinical samples based on chromosome 7 gains.** (**A**). Neutrophil quantification within the on-PD-1 inhibitor samples (lesion 2) comparing sites by chromosome 7 copy number status as determined from MCP counter (left) and CIBERSORT (right). (**B**) Neutrophil content derived from CIBERSORT within TCGA SKCM samples grouped by chromosome 7 copy number status. (**C**). KEGG pathways enriched in melanoma samples harboring chromosome 7 gains revealed numerous pathways involved in reaction to bacterial infections, phagosome and lysosome formation, and antigen processing, consistent with the highly neutrophil activation-dominant GO term enrichments observed. (**D-F**) Neutrophil content derived from CIBERSORT within clinical immune checkpoint blockade-treated samples grouped by reported response status (NR=non-responder, R=responder) from the Van Allen anti-CTLA-4 cohort (**D**) and the Hugo (**E**) and Riaz (**F**) anti-PD-1 cohorts.

17
