# Supplementary material for: Spatially resolved analyses link genomic and immune diversity and reveal unfavorable neutrophil activation in melanoma
Source: Nat Commun. 2020 Apr 15;11:1839. doi: 10.1038/s41467-020-15538-9 (PMC7160105; doi:10.1038/s41467-020-15538-9)
Supplement: Supplementary file 3 — Description of Additional Supplementary Files [file 41467_2020_15538_MOESM3_ESM.pdf]

Description of Additional Supplementary Files

File Name: Supplementary Data 1
Description: Intra-tumoral heterogeneity sample analysis modalities by region.

File Name: Supplementary Data 2
Description: List of genes included on the targeted mutation panel.

File Name: Supplementary Data 3
Description: Somatic point-mutation events identified by targeted gene sequencing across multiple sub-regions of a melanoma tumor reveal significant intra-tumoral mutational heterogeneity.

File Name: Supplementary Data 4
Description: Differentially-expressed genes comparing chromosome 10 loss versus stable regions.

File Name: Supplementary Data 5
Description: Complete list of differentially-expressed genes confirming the activity of multiple immune subsets in regions of heavy immune infiltration.

File Name: Supplementary Data 6
Description: CIBERSORT-derived immune cell population frequencies within each sampled sub-region of the on-PD-1 inhibitor tumor.

File Name: Supplementary Data 7
Description: List of neutrophil marker genes used by immune deconvolution algorithms.

File Name: Supplementary Data 8
Description: Neutrophil-related Gene Ontology (GO) term sets.
