# Supplementary material for: Spatially resolved analyses link genomic and immune diversity and reveal unfavorable neutrophil activation in melanoma
Source: Nat Commun. 2020 Apr 15;11:1839. doi: 10.1038/s41467-020-15538-9 (PMC7160105; doi:10.1038/s41467-020-15538-9)
Supplement: Supplementary file 12 — Reporting Summary [file 41467_2020_15538_MOESM12_ESM.pdf]

nature research

Corresponding author(s): P. Andrew Futreal

Last updated by author(s): Jan 25, 2020

Reporting Summary

Nature Research wishes to improve the reproducibility of the work that we publish. This form provides structure for consistency and transparency in reporting. For further information on Nature Research policies, see Authors & Referees and the Editorial Policy Checklist .

Statistics

For all statistical analyses, confirm that the following items are present in the figure legend, table legend, main text, or Methods section.

n/a	Confirmed	
☐	☒	The exact sample size (n) for each experimental group/condition, given as a discrete number and unit of measurement
☐	☒	A statement on whether measurements were taken from distinct samples or whether the same sample was measured repeatedly
☐	☒	The statistical test(s) used AND whether they are one- or two-sided<br>*Only common tests should be described solely by name; describe more complex techniques in the Methods section.*
☐	☒	A description of all covariates tested
☐	☒	A description of any assumptions or corrections, such as tests of normality and adjustment for multiple comparisons
☒	☐	A full description of the statistical parameters including central tendency (e.g. means) or other basic estimates (e.g. regression coefficient) AND variation (e.g. standard deviation) or associated estimates of uncertainty (e.g. confidence intervals)
☐	☒	For null hypothesis testing, the test statistic (e.g. F, t, r) with confidence intervals, effect sizes, degrees of freedom and P value noted<br>*Give P values as exact values whenever suitable.*
☒	☐	For Bayesian analysis, information on the choice of priors and Markov chain Monte Carlo settings
☒	☐	For hierarchical and complex designs, identification of the appropriate level for tests and full reporting of outcomes
☐	☒	Estimates of effect sizes (e.g. Cohen's d, Pearson's r), indicating how they were calculated

Our web collection on statistics for biologists contains articles on many of the points above.

Software and code

Policy information about availability of computer code

Data collection	Patient data, tumor samples, and matched peripheral blood leukocyte samples were obtained and used in accordance with research protocols approved by the local Institutional Review Board of the University of Texas MD Anderson Cancer Center. Biospecimens were retrieved, collected and analysed under UT MD Anderson Cancer Center Institutional Review Board approved protocols in accordance with the Declaration of Helsinki.<br>Sequencing data (whole exome sequencing, RNA sequencing, T200 targeted gene panel sequencing) have been deposited in the European Genome-Phenome Archive under accession EGAS000001003292. Other transcriptomic datasets analyzed in this study can be retrieved from dbGAP under the accession dbGaP phs000452.v2.p1 for the Van Allen dataset and from the GEO repository under the accessions GSE78220 for the Hugo dataset and GSE91061 for the Riaz dataset The TCGA melanoma dataset can be accessed on the GDC portal (portal.gdc.cancer.gov, cohort TCGA SKCM)
Data analysis	Statistical analyses were performed using R v3.5.095. Analysis packages and tools used are described in the relevant methods sections and tabulated in the Key Resources Table. Statistical tests included Welch's two sample t-test and Spearman's rank correlation with the Benjamin Hochberg correction for an adjusted p-value of 0.05. The R package plot3D and Plotly was used to map sequencing-derived data to spatial locations. Data was parsed and organized through R packages tidyr, reshape2 and dplyr. Dendograms and tanglegrams were constructed using dendextend. Plotting was done through ggplot2 and ggrepel.

For manuscripts utilizing custom algorithms or software that are central to the research but not yet described in published literature, software must be made available to editors/reviewers. We strongly encourage code deposition in a community repository (e.g. GitHub). See the Nature Research guidelines for submitting code & software for further information.

Data

Policy information about availability of data

All manuscripts must include a data availability statement. This statement should provide the following information, where applicable:

- Accession codes, unique identifiers, or web links for publicly available datasets
- A list of figures that have associated raw data
- A description of any restrictions on data availability

Patient data, tumor samples, and matched peripheral blood leukocyte samples were obtained and used in accordance with research protocols approved by the local Institutional Review Board of the University of Texas MD Anderson Cancer Center. Biospecimens were retrieved, collected and analysed under UT MD Anderson Cancer Center Institutional Review Board approved protocols in accordance with the Declaration of Helsinki.
Sequencing data (whole exome sequencing, RNA sequencing, T200 targeted gene panel sequencing) have been deposited in the European Genome-Phenome Archive under accession EGAS000001003292. Other transcriptomic datasets analyzed in this study can be retrieved from dbGAP under the accession dbGaP phs000452.v2.p1 for the Van Allen dataset and from the GEO repository under the accessions GSE78220 for the Hugo dataset and GSE91061 for the Riaz dataset The TCGA melanoma dataset can be accessed on the GDC portal (portal.gdc.cancer.gov, cohort TCGA SKCM)

Field-specific reporting

Please select the one below that is the best fit for your research. If you are not sure, read the appropriate sections before making your selection.

☒ Life sciences ☐ Behavioural & social sciences ☐ Ecological, evolutionary & environmental sciences

For a reference copy of the document with all sections, see nature.com/documents/nr-reporting-summary-flat.pdf

Life sciences study design

All studies must disclose on these points even when the disclosure is negative.

Sample size	No sample size calculation was performed.
Data exclusions	No data exclusions were recorded.
Replication	All methods for reproducing the data have been indicated in the methods.
Randomization	Not relevant.
Blinding	Not relevant.

Reporting for specific materials, systems and methods

We require information from authors about some types of materials, experimental systems and methods used in many studies. Here, indicate whether each material, system or method listed is relevant to your study. If you are not sure if a list item applies to your research, read the appropriate section before selecting a response.

Materials & experimental systems

n/a	Involved in the study	
☐	☒	Antibodies
☒	☐	Eukaryotic cell lines
☒	☐	Palaeontology
☒	☐	Animals and other organisms
☐	☒	Human research participants
☐	☒	Clinical data

Methods

n/a	Involved in the study	
☒	☐	ChIP-seq
☒	☐	Flow cytometry
☒	☐	MRI-based neuroimaging

Antibodies

Antibodies used

Immunohistochemistry (IHC) was performed on each of the 4 FFPE sections using an automated stainer (Leica Bond Max, Leica Biosystems) using primary antibodies against SOX10 (polyclonal, 1:50, Cell Marque, Cat. No. 383A-7), CD45-LCA (clones 2B11 + PD7/26, 1:300, Dako, Cat. No. M0701), CD45-RO (clone UCHL1, undiluted, Leica Biosystems, Cat. No. PA0146), CD4 (clone 4B12, 1:80, Leica Biosystems, Cat. No. NCL-L-CD4-368), CD8α (clone C8/144B, 1:25, ThermoScientific, Cat. No. MA5-13473), Granzyme B (clone GrB-7, 1:25, ThermoScientific, Cat. No. MA1-35461), FoxP3 (clone 206D, 1:50, BioLegend, Cat. No. 320102), LAG-3 (clone D2G4O, 1:100, Cell Signaling Technology, Cat. No. 15372), PD-1 (clone EPR4877(2), 1:250, Abcam, Cat. No. ab137132), PD-L1 (clone E1L3N, 1:100, Cell Signaling Technology, Cat. No. 13684), PAX5 (clone 1EW, undiluted, Leica Biosystems, Cat. No. PA0552), CD68 (clone PG-M1, 1:450, Dako, Cat. No. M0876), CD57 (clone HNK1/Leu-7, 1:250, Abcam, Cat. No. ab187274), and phospho-p44/42(Erk1/2)(Thr202/Tyr204) (clone D13.14.4E, 1:300, Cell Signaling Technology, Cat. No. 4370). Slides were

	counter-stained with hematoxylin, scanned using an Aperio slide scanner (Aperio AT Turbo, Leica Biosystems) and digitized images analyzed using the Aperio ImageScope software (Aperio - Leica Biosystems). Three-dimensional reconstruction re-connecting the frozen and FFPE slices in a sequential order was performed based on the documented inter-slice relationships and histological findings. The IHC slices were gridded into smaller pieces in the ImageScope software to match the gridding of frozen sections, and the results were obtained for each sub-region. IHC-derived cell subset results were quantified as the number of positive-staining cells for each antibody per mm2, using custom-tuned algorithms based on nuclear v9, membrane v9, or cytoplasmic v1 algorithms as appropriate for the staining pattern of each antibody.
Validation	All antibodies under consideration have been validated.

Human research participants

Policy information about studies involving human research participants

Population characteristics	Patient under investigation includes a Caucasian female diagnosed with metastatic melanoma.
Recruitment	Not applicable
Ethics oversight	Indicated in manuscript

Note that full information on the approval of the study protocol must also be provided in the manuscript.

Clinical data

Policy information about clinical studies

All manuscripts should comply with the ICMJEguidelines for publication of clinical research and a completedCONSORT checklist must be included with all submissions.

Clinical trial registration	Not applicable
Study protocol	Indicated in manuscript
Data collection	Indicated in manuscript
Outcomes	Not applicable
